# Diversity of MHC IIB genes and parasitism in hybrids of evolutionarily divergent cyprinoid species indicate heterosis advantage

**DOI:** 10.1038/s41598-021-96205-x

**Published:** 2021-08-19

**Authors:** Andrea Šimková, Lenka Gettová, Kristína Civáňová, Mária Seifertová, Michal Janáč, Lukáš Vetešník

**Affiliations:** 1 Department of Botany and Zoology, Faculty of Science, Masaryk University, Kotlářská 2, 611 37 Brno, Czech Republic; 2 Institute of Vertebrate Biology, Academy of Sciences of the Czech Republic, Květná 8, 603 65 Brno, Czech Republic

**Keywords:** Evolutionary ecology, Evolution

## Abstract

The genes of the major histocompatibility complex (MHC) are an essential component of the vertebrate immune system and MHC genotypes may determine individual susceptibility to parasite infection. In the wild, selection that favors MHC variability can create situations in which interspecies hybrids experience a survival advantage. In a wild system of two naturally hybridizing leuciscid fish, we assessed MHC IIB genetic variability and its potential relationships to hosts’ ectoparasite communities. High proportions of MHC alleles and parasites were species-specific. Strong positive selection at specific MHC codons was detected in both species and hybrids. MHC allele expression in hybrids was slightly biased towards the maternal species. Controlling for a strong seasonal effect on parasite communities, we found no clear associations between host-specific parasites and MHC alleles or MHC supertypes. Hybrids shared more MHC alleles with the more MHC-diverse parental species, but expressed intermediate numbers of MHC alleles and positively selected sites. Hybrids carried significantly fewer ectoparasites than either parent species, suggesting a hybrid advantage via potential heterosis.

## Introduction

Reciprocal interspecific hybrids are often characterized by high vigour resulting from heterosis^1,2^. Hybrid advantage usually reflects the superiority of F1 hybrids over one or both parents for traits related to development, growth, maintenance, and resistance to environmental factors and diseases (e.g.^3–5^). In line with the hybrid advantage hypothesis, fish hybrids often exhibit higher potential to survive, faster growth, and better condition status when compared to parental species (e.g.^6–8^). In spite of the high frequency of hybridization in fish and especially in highly diversified Cyprinoidei^9^, the potential of immune resistance/susceptibility has not been comprehensively studied in fish hybrids coexisting in natural habitats with their parental species.

In hybrids of wild living vertebrates, selection may favor the variability of immune genes associated with high resistance to parasites, and potentially interspecies hybrids may through the variability of immune genes exhibit a survival advantage over parents. The major histocompatibility complex (MHC) includes the functional immune genes recognized as an essential component of the vertebrate adaptive immune system (although MHC is absent in jawless fish). Thus, MHC may represent appropriate candidate of immune genes when investigating hybrid heterosis.

A characteristic feature of MHC genes is trans-species polymorphism, i.e. the passage of allelic lineages from ancestral to descendant species. This polymorphism was previously documented in cyprinoids^10,11^. In teleost fish (as well as in other jawed vertebrates), two groups of classical MHC genes—class I and class II have been recognized and described. Whilst MHC class I molecules predominantly bind peptides derived from intracellular pathogens (viruses and bacteria), MHC class II molecules bind peptides derived from extracellular pathogens (especially macroparasites)^12,13^. MHC molecules play a central role in parasite recognition and elimination. The binding between MHC molecules and foreign peptides derived from parasites is realized by a small number of amino acid residues of the peptide-binding regions (PBR)^13–15^. Therefore, parasite-mediated selection is considered as one of the main drivers of the evolution and maintenance of high MHC polymorphism^14,16–19^, and thus, the MHC provides a genetic basis for the adaptation of vertebrate hosts to coevolving parasites. Using a theoretical model, an intermediate number of MHC alleles was postulated as the optimal MHC for an individual^20^. Based on this model, the maximum number of MHC alleles is disadvantageous because it results in the presentation of more self-peptides with the subsequent elimination of self-reactive T-cells in individuals. In the study of three-spined stickleback, an intermediate number of MHC IIB alleles was associated with minimal parasite load at the individual level^21^. However, the super-optimal individual diversity of MHC genes in hybrids was hypothesized, with the expectation that hybrids with super-optimal MHC diversity should suffer more from parasites^22^. This mechanism should be very effective in selecting against interspecies hybrids. Concerning wild-living vertebrates, the genetic variation in MHC genes between hybridizing species was studied, and adaptive MHC introgression was documented, especially in newts^23–25^. The mechanisms generating MHC diversity in hybrid zones were also investigated in fishes^26^ focusing on hybrid zones of native and endemic *Parachondrostoma toxostoma* and invasive *Chondrostoma nasus* (Leuciscidae) in Southern France. Bidirectional gene flow for MHC IIB genes was shown. The authors reported the expression of an intermediate number of MHC IIB alleles in hybrids of the first generation (F1 hybrids), representing their potential advantage; however, hybrids expressed a higher proportion of MHC genes of more genetically variable species, i.e. native *P. toxostoma*. Higher MHC similarity between genetically more MHC-variable species and hybrids was proposed to explain the low susceptibility of native *P. toxostoma* and hybrids to ectoparasitic monogeneans widely infecting non-native and genetically less diverse *C. nasus*^26^. Innate and adaptive immune-relevant factors can be transferred maternally, some of them functioning in the defense of fish larvae against pathogens^27^. However, paternal effects have also been reported for innate immunity^28^. This may evoke the question of whether the direction of genetic introgression affects hybrid immunocompetence and susceptibility to parental species-specific parasites.

Evolutionary divergence is an important mechanism affecting genetic incompatibilities between species and determining the successfulness of hybrids. The hybridization between common bream (*Abramis brama*) (henceforth, ‘bream’) and roach (*Rutilus rutilus*), two evolutionarily divergent leuciscid species, has been widely documented^29–32^. Whilst a strong bias toward hybrids with bream maternal ancestry was documented in some regions^32–34^, similar proportions of hybrids with bream and roach maternal inheritance were documented in other regions^35^. Hybrids of bream and roach in natural habitats are primarily produced as F1 crosses of parental species, and the presence of post-F1 hybrids is negligible^31–33^ indicating that F1 hybrids experience fitness disadvantages when compared with pure species. In some regions, all hybrids of bream and roach sampled in natural habitats are identified as solely F1 crosses^34,35^. F1 hybrids of these leuciscid species express intermediate phenotypic and ecological traits^32,34^, utilization of a broader trophic spectrum and high tolerance to fluctuations of food supply compared to parental species^31,36^. Hybrid advantage was documented in terms of high survival at early developmental stages^37^, fast growth^38^, and low susceptibility to parasites that are host-specific to bream or host-specific to roach^35^.

The aim of the present study was to analyze the variability of expressed MHC genes in natural populations of two coexisting and evolutionarily divergent leuciscid species, *A. brama* and *R. rutilus*, and their reciprocal hybrids (F1 generation). In accordance with previous studies in hybrid zones of cyprinoids^26^, we hypothesized that the intermediate variability of MHC genes in hybrids, measured by the number of *DAB* alleles, is associated with low parasite load (lower parasite load in F1 hybrids of bream x roach when compared to parental species was previously shown^35^). We also hypothesized some similarity in MHC profiles between hybrids and each of the parental species, on the basis that hybrids may harbor the specific parasites of both parental species in cyprinoid hosts^35,39^. Finally, based on the prediction that MHC is involved in recognition of parasites, we hypothesized the potential associations between specific MHC alleles (present only in one parental species and potentially in hybrids) and specific parasite species (also present only in one parental species and potentially in hybrids). We also investigated potential functional associations between MHC (using MHC supertypes) and parasites.

## Results

### MHC diversity and positive selection

The amplification of cDNA representing expressed *DAB* genes was successful in 94% of analyzed specimens (Table 1). The overall number of expressed *DAB1* alleles was lower than the number of expressed *DAB3* alleles in all three groups of fish. Numbers of specimens for which the expression of only the alleles of *DAB1* genes, only the alleles of *DAB3* genes and the expression of the alleles of both *DAB1* and *DAB3* genes were reported, are shown in Table 1. Most roach specimens expressed the alleles of both *DAB1* and *DAB3* genes, whilst most bream and hybrids expressed the alleles of *DAB3* genes only.

**Table 1:** Number of specimens for *A. brama*, *R. rutilus* and F1 hybrids with the expression of *DAB* alleles, N is population size.

	N	No *DAB* alleles	Only *DAB1* alleles	Only *DAB3* alleles	*DAB1* and *DAB3* alleles
*A. brama*	109	7	3	80	19
*R. rutilus*	97	3	15	34	45
Hybrids	88	9	8	46	25

The total number of *DAB* alleles per fish group, total number of private alleles, and number of alleles per individual are shown in Table 2. Roach were more polymorphic than bream, and hybrids were intermediate. However, the overall number of *DAB1* alleles was similar in bream and hybrids and was lower when compared to roach (Table 2). Hybrids shared five *DAB1* alleles with roach and only one *DAB1* allele with bream. A single *DAB1* allele was shared among all three groups of fish. Hybrids shared 13 *DAB3* alleles with roach and 5 *DAB3* alleles with bream; another five *DAB3* alleles were shared among all three groups of fish.

**Table 2:** Number of *DAB* alleles in *A. brama*, *R. rutilus* and F1 hybrids.

Population	DAB alelles	Private *DAB* alleles	*DAB* alleles per individual			Microsatetellite allelic richnes
	(*DAB1*, *DAB3*)	(*DAB1*, *DAB3*)	*DAB1*	*DAB3*	Overall (mean ± S.D.)	(mean ± S.D.)
*A. brama*	30 (6, 24)	18 (4, 14)	0–1	0–2	1–3 (1.58 ± 0.62)	1.68 ± 1.06
*R. rutilus*	65 (16, 49)	41 (10, 31)	0–3	0–4	1–5 (1.83 ± 0.89)	3.13 ± 1.84
Hybrids	46 (7, 39)	16 (0, 16)	0–2	0–3	1–4 (1.71 ± 0.72)	3.91 ± 1.57

The overall number of *DAB* alleles is calculated for fish which expressed at least one *DAB* allele. Microsatellite allelic richness is also shown.

**Figure 1:**
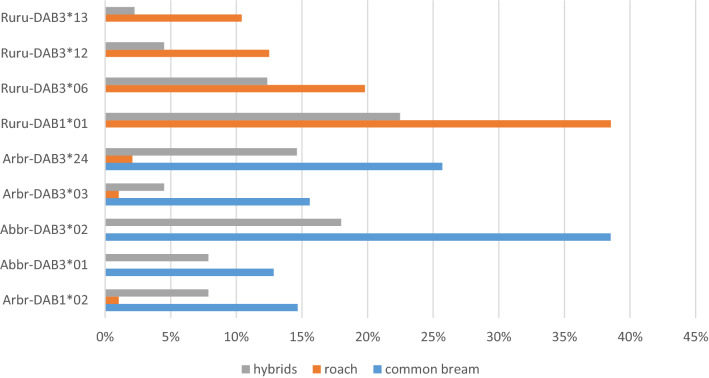
The frequencies of the most common *DAB* alleles in common bream, roach and their F1 hybrids. Note that only the frequencies of the most frequent alleles for common bream (5 alleles) and roach (4 alleles) are shown.

Different alleles were reported as the most frequent for bream and roach populations (Fig. 1). Bream frequently expressed two specific alleles (found solely in bream and hybrids) and three other alleles shared with hybrids and roach. Roach most frequently expressed four specific alleles. Other alleles in bream and roach were present at frequencies ≤ 6%. Hybrids most frequently expressed the common alleles of parental species (two roach-specific alleles and four alleles frequently reported in bream). Other alleles in hybrids were present at frequencies < 6%. Hybrid-specific alleles were present at frequencies < 3.4%.The hybrids with bream in maternal position and the hybrids with roach in maternal position expressed some of the common alleles in different frequency (Supplement S1).

The mean number of *DAB* alleles per hybrid individual, as well as the maximum number of alleles in hybrid individuals, was intermediate between those of bream and roach (Table 2). A significant difference in the number of *DAB1* alleles was found among fish groups (Kruskal–Wallis H-test, *p* < 0.001). Hybrids expressed an intermediate number of *DAB1* alleles between bream and roach; multiple comparisons revealed significant differences between bream and roach (*p* < 0.001) and between hybrids and each of the parental species (*p* < 0.05). The Kruskal–Wallis H-test also revealed a significant difference in the total number of *DAB3* alleles among bream, roach, and hybrids (*p* = 0.015). Even though the hybrids tended to express the intermediate number of *DAB3* alleles, multiple comparisons revealed the significant difference only between bream and roach (*p* = 0.036).

**Figure 2:**
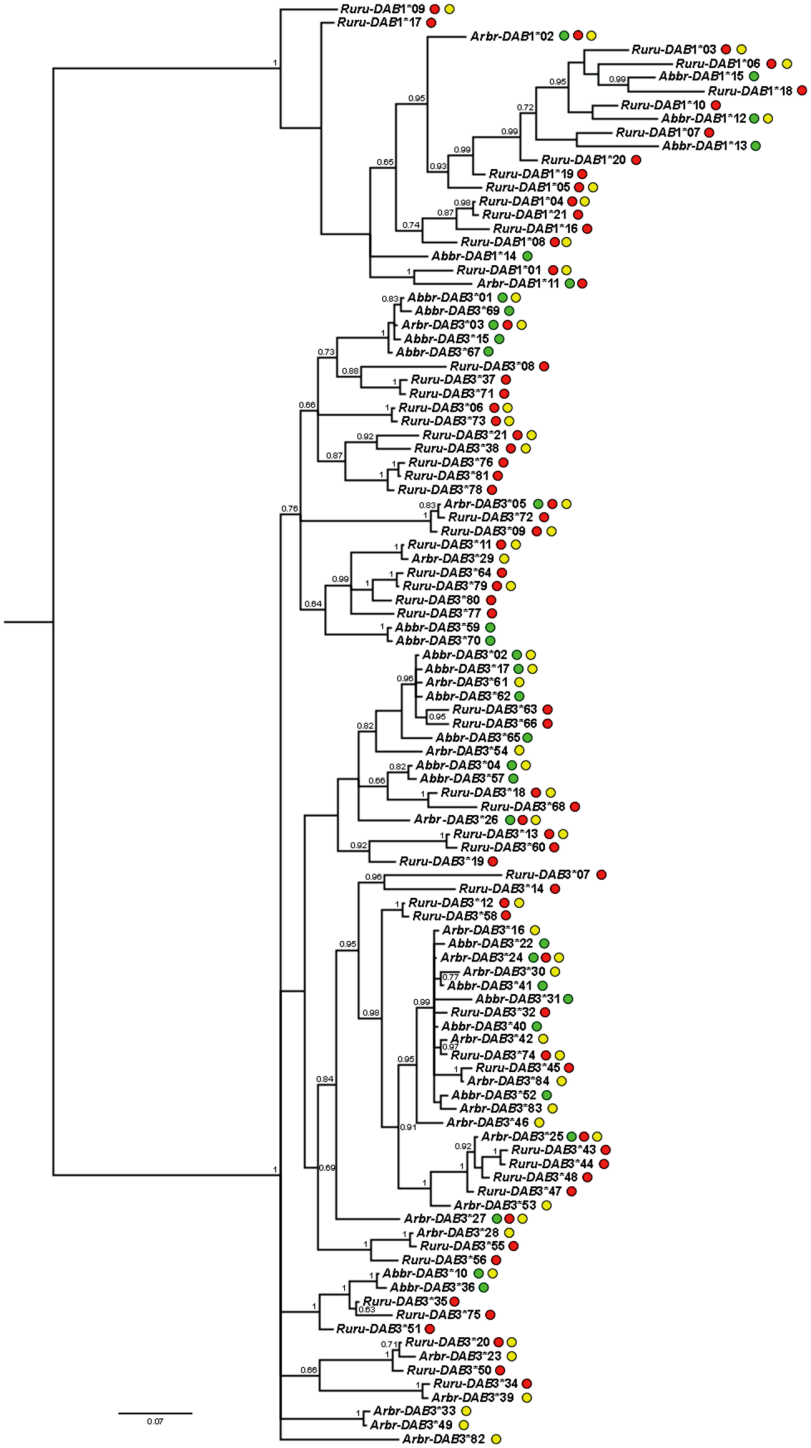
The Bayesian inference tree including *DAB1* and *DAB3* alleles identified in *A. brama*, *R. rutilus* and their hybrids. Numbers along branches represent posterior probabilities (> 0.60) resulting from BI. Green dots indicate the alleles present in *A. brama,* red dots indicate the alleles present in *R. rutilus*, and yellow dots indicate the alleles present in hybrids. *DAB* alleles were abbreviated as follows: *Abbr* present solely in *A. brama* or both *A. brama* and hybrids*,* *Ruru* present solely in *R. rutilus* or both *R. rutilus* and hybrids, *Arbr* alleles present in both parental species and hybrids, or alternatively present solely in hybrids.

Phylogenetic reconstruction (Fig. 2) showed that *DAB* alleles clustered in two main lineages, i.e. the lineage of *DAB1* alleles and the lineage of *DAB3* alleles. Even if some clusters tended to include mostly roach alleles and others mostly bream alleles, many clusters included the alleles of both species, which supported trans-species polymorphism. A few alleles shared by both species and their hybrids were situated randomly within a phylogenetic tree.

**Figure 3:**
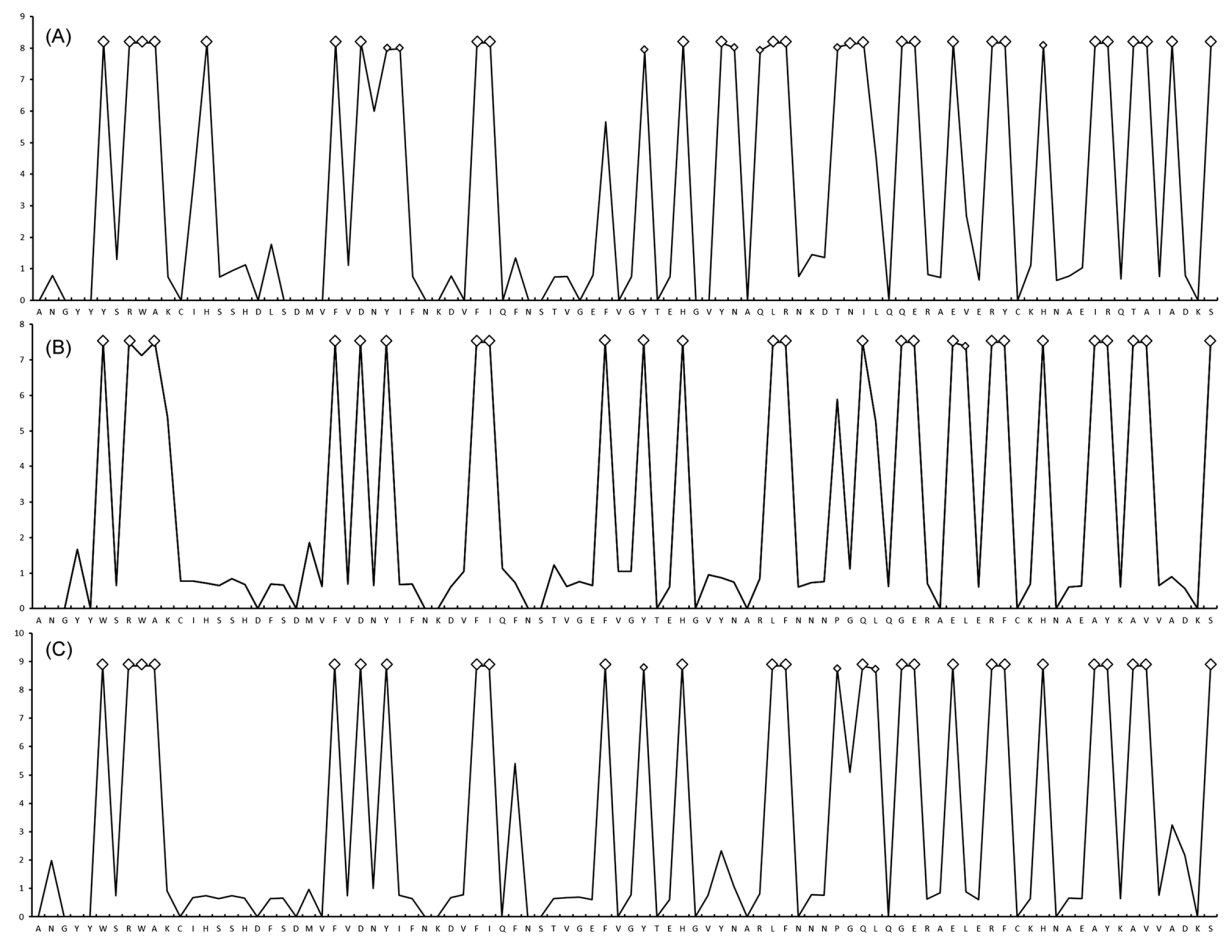
Approximate posterior means of ω calculated as the weighted average of ω over the 11 site classes and weighted by the posterior probabilities under the M8 site-model are shown for *DAB* sequence variants for (**A**) *A. brama*, (**B**) *R. rutilus*, and (**C**) their respective hybrids. Sites inferred to be under positive selection at the 99% level are indicated by large white squares and those at the 95% level are indicated by small white squares.

The likelihood ratio statistic comparing the two models—one not incorporating selection and one incorporating selection (M1a versus M2a, M0 versus M3, M7 versus M8)—indicated that the models that accounted for the sites under selection (i.e. M2a, M3 and M8) fit the data significantly better (*p* < 0.001) than simpler models that did not allow for selection (i.e. M1a, M0 and M7), which indicates a signal of positive selection at specific sites in *DAB* sequences. Log-likelihood values and parameter estimates under random-site models are shown in Supplement S2. On the basis of BEB analysis, 24 and 33 PSS in bream were identified using the M2a and M8 models respectively, whilst 25 sites using the M2a model and 26 sites using the M8 model were identified under positive selection in roach. The Bayes identification of sites under positive selection calculated using the M8 model is included in Fig. 3. The same 24 PSS under the M8 model were identified in both species. In addition, next 9 codons only in bream and 2 codons only in roach were identified under positive selection. The pattern of PSS distribution in hybrids was intermediate between bream and roach. The 24 PSS shared by bream and roach were also identified in hybrids. In addition, one codon under positive selection was shared by both bream and hybrids, and one codon under positive selection was shared by roach and hybrids. A single codon was identified under positive selection solely in hybrids.

### Associations between MHC genes and parasites

**Figure 4:**
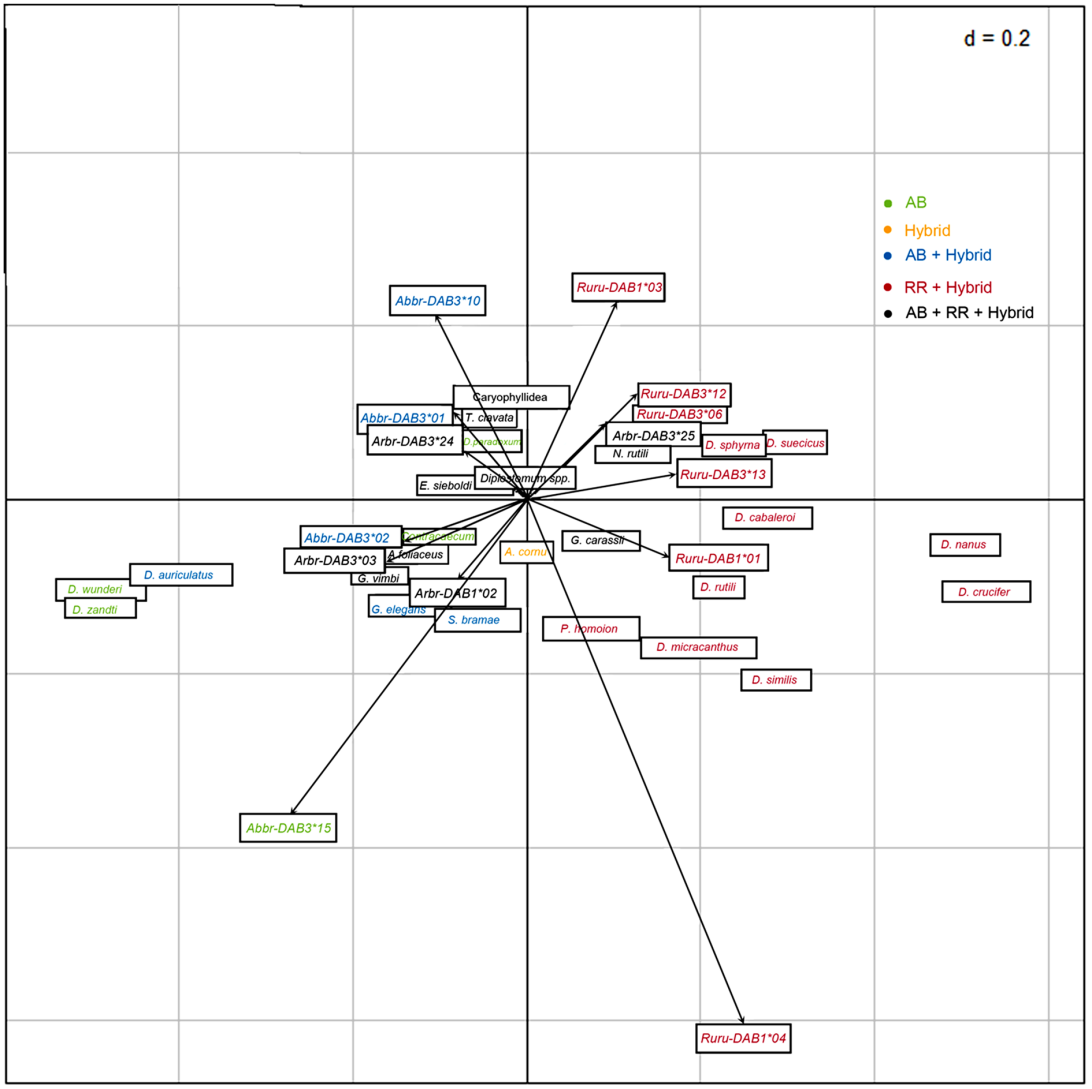
Co-inertia analysis (COIA) of the MHC genetic variation (MHC alleles data) and metazoan parasite species of common bream, roach and hybrids. Color labelling of *DAB* alleles and parasites corresponds to their presence in different fish groups or shared groups.

Parasite data for fish analysed for MHC diversity are included in Table 3. No significant covariance between MHC alleles and parasite species data or parasite groups data was found using COIA for each of bream, roach, and hybrids separately (*p* > 0.05). No significant covariance between MHC alleles and parasite groups was found when including all fish groups into analysis (*p* > 0.05) (Supplement S3). Similarly, no significant covariance between MHC supertypes and parasite groups data (Supplement S4) was found when including all fish in the analyses or when analyzing common bream, roach or hybrids separately (*p* > 0.05). When COIA was performed including MHC alleles and all fish groups (bream, roach, and hybrids), the *DAB* alleles and metazoan parasite species exhibited significant covariance in the COIA model (RV = 0.16, *p* < 0.003). The two first axes of the COIA (F1 and F2) accounted for 75.05% of the total variance shared between the two matrices (F1 65.99% and F2 9.06%). Comparison between the distribution of parasitological and genetic variables on the COIA factor maps revealed species-specific associations (Fig. 4). Correspondence analysis row scores on F1 separated bream and roach individuals on the basis of their specific MHC alleles and host-specific parasite species (Supplement S5).

**Table 3:** Parasite infection in *A. brama*, *R. rutilus* and hybrids.

	*A. brama*			*R. rutilus*			Hybrids		
	A	II	P	A	II	P	A	II	P
**Total Monogenea**	94.98 ± 90.04	515	98	57.25 ± 51.72	248	99	9.10 ± 10.59	67	94
*Dactylogyrus crucifer*	–	–	–	39.34 ± 37.53	185	96	3.72 ± 5.26	33	76
*Dactylogyrus caballeroi*	–	–	–	3.10 ± 6.82	41	45	0.16 ± 0.71	6	9
*Dactylogyrus nanus*	–	–	–	7.81 ± 8.12	35	83	1.96 ± 3.08	17	58
*Dactylogyrus suecicus*	–	–	–	2.76 ± 4.39	25	70	1.92 ± 3.73	25	49
*Dactylogyrus similis*	–	–	–	1.22 ± 2.93	19	35	0.25 ± 0.71	3	12
*Dactylogyrus sphyrna*	–	–	–	0.21 ± 0.62	3	15	0.11 ± 0.41	2	8
*Dactylogyrus micracanthus*	–	–	–	0.14 ± 0.47	3	9	0.10 ± 0.34	2	9
*Dactylogyrus rarissimus*		–	–	0.02 ± 0.14	1	2	–	–	–
*Dactylogyrus rutili*	–	–	–	0.20 ± 0.49	2	16	0.04 ± 0.21	1	4
*Dactylogyrus fallax*	–	–	–	0.02 ± 0.14	1	2	0.01 ± 0.11	1	1
*Dactylogyrus auriculatus*	56.26 ± 68.25	303	70	–	–	–	0.12 ± 0.45	3	9
*Dactylogyrus wunderi*	16.69 ± 14.48	66	94	–	–	–	0.01 ± 0.11	1	1
*Dactylogyrus zandti*	16.25 ± 14.24	69	94	–	–	–	–	–	–
*Gyrodactylus vimbi*	3.35 ± 12.68	79	13	0.54 ± 2.44	19	10	0.08 ± 0.31	2	7
*Gyrodactylus carassii*	0.04 ± 0.30	3	2	0.98 ± 7.67	75	10	0.01 ± 0.11	1	1
*Gyrodactylus elegans*	1.45 ± 10.29	106	17	–	–	–	0.09 ± 0.32	2	8
*Diplozoon paradoxum*	0.04 ± 0.19	1	4	–	–	–	–	–	–
*Paradiplozoon homoion*	–	–	–	0.04 ± 0.25	2	3	0.07 ± 0.29	2	6
**Total Crustacea**	7.24 ± 12.35	81	87	0.76 ± 1.53	9	32	8.71 ± 13.48	58	76
*Argulus foliaceus*	2.98 ± 6.50	41	46	0.52 ± 1.42	9	20	1.12 ± 2.71	20	38
*Ergasilus sieboldi*	4.26 ± 9.94	72	75	0.24 ± 0.68	5	17	7.58 ± 12.59	58	67
**Total Digenea**	1.92 ± 5.86	55	41	1.19 ± 2.39	13	39	5.27 ± 10.28	61	61
*Diplostomum* spp.	1.10 ± 2.44	17	36	0.85 ± 1.86	11	31	1.43 ± 2.46	10	43
*Tylodelphys clavata*	0.69 ± 5.29	54	5	0.33 ± 1.14	7	11	3.48 ± 9.87	61	33
*Sphaerostoma bramae*	0.13 ± 0.77	7	4	–	–	–	0.05 ± 0.42	4	1
*Apharyngostrigea cornu*	–	–	–	–	–	–	0.31 ± 1.82	14	3
**Total Acanthocephala**	0.05 ± 0.34	3	2	0.35 ± 1.66	14	10	0.04 ± 0.33	3	2
*Neoechinorhynchus rutili*	0.05 ± 0.34	3	2	0.35 ± 1.66	14	10	0.04 ± 0.33	3	2
**Total Cestoda**	0.76 ± 2.90	20	15	0.61 ± 1.81	11	17	0.15 ± 0.55	4	9
Caryophyllaeidae spp.	0.74 ± 2.89	20	14	0.61 ± 1.81	11	17	0.15 ± 0.55	4	9
*Ligula intestinalis*	0.02 ± 0.19	2	1	–	–	–	–	–	–
**Total Nematoda**	0.14 ± 0.54	4	8	–	–	–	0.02 ± 0.15	1	2
*Contracaecum* sp.	0.14 ± 0.54	4	8	–	–	–	0.02 ± 0.15	1	2

*A* abundance (mean ± S.D.), *II* maximum intensity of infection per infected fish specimen, *P* prevalence (proportion of infected fish within whole sample, in %).

**Figure 5:**
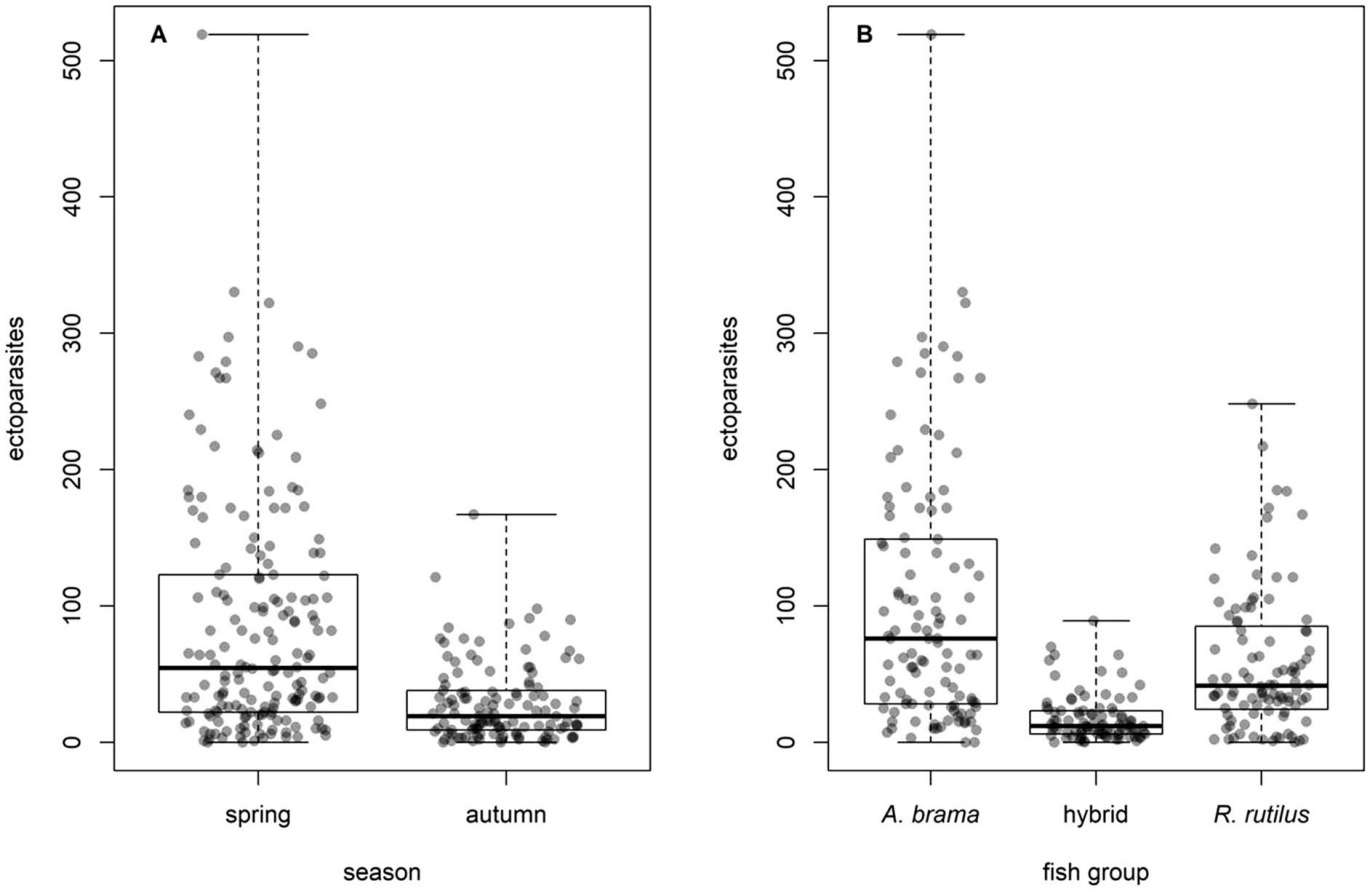
Effects of season (**A**) and fish group (**B**) on ectoparasite abundance.

The total number of alleles, number of *DAB1* alleles, or number of *DAB3* alleles appeared to have no or very limited effect on parasite abundance/prevalence, as this effect was significant only in three out of 26 GLMM models testing the effects of season, fish group and number of alleles on parasite load. The effects of season and species were more pronounced, being present in the final models for most of the taxonomical groups (Table 4, see Fig. 5 for ectoparasites). Generally, fish were more parasitized in spring than in autumn, both in terms of prevalence, abundance and richness. Whilst roach harboured higher *Dactylogyrus* species richness when compared to bream and hybrids, bream reached higher total parasite abundance, ectoparasite abundance, monogenean abundance, and *Dactylogyrus* abundance. Bream and hybrids reached a higher abundance of Crustacea when compared to roach, and hybrids themselves also reached a higher abundance of Digenea when compared to bream and roach. For the significance of the abovementioned differences see Supplement S6.

**Table 4:** Terms remaining in final models originating by backward stepwise regression from GLMMs detecting effects of season, fish group and number of alleles for 13 response variables (richness, abundance or, in case of prevalence ≤ 30%, prevalence was used instead of abundance).

Response	Total number of alleles			Number of DAB1 and DAB3 alleles		
	Term	P	AICc	Term	P	AICc
Total richness^1^	Season	< 0.001	60.8			
*Dactylogyrus* richness^1^	Season	< 0.001	22.2			
	Fish group	< 0.001	29.7			
Total abundance^2^	Season	< 0.001	83.4			
	Fish group	< 0.001	103.3			
Ectoparasite abundance^2^	Season	< 0.001	133.4			
	Fish group	< 0.001	155.4			
Endoparasite abundance^2^	Season	< 0.001	18.1	**Season**	< 0.001	17.3
	Fish group	< 0.001	11	**DAB1_t : fish group**	0.033	2.6
Monogenea abundance^2^	Season	< 0.001	65.6			
	Fish group	< 0.001	172.7			
Crustacea abundance^3^	Season	< 0.001	21.2			
	Fish group	< 0.001	95.8			
Digenea abundance^3^	Season	0.021	3.2			
	Fish group	< 0.001	17.4			
Acanthocephala prevalence^4^	Season	< 0.001	14.4			
	Fish group	0.020	3.7			
Nematoda prevalence^4^	Fish group	0.002	8.8	**Fish group**	0.011	4.4
				**DAB1_t**	0.023	3.5
Cestoda prevalence^4^	Season	< 0.001	20.6			
*Dactylogyrus* abundance^3^	Season	< 0.001	59.8			
	Fish group	< 0.001	168.3			
*Gyrodactylus* prevalence^4^	Season	< 0.001	46.4	**season**	< 0.001	45.4
	Fish group	0.014	4.4	**DAB3_t : fish group**	0.034	2.6

For each term, AICc and P of log-likelihood test for the term removal are shown. For models detecting effect of number of *DAB1* and *DAB3* alleles, only final models different from those using total number of alleles as predictor are shown (i.e. the significant effects of season and fish group are not shown in table for the models including number of *DAB1* and *DAB3* alleles). See methods for full model formulae. Terms of models where number of alleles played a role are in bold. Response distribution as specified in the model: ^1^Poisson, ^2^negative binomial, ^3^Poisson with observation level random effects, ^4^Bernoulli.

**Figure 6:**
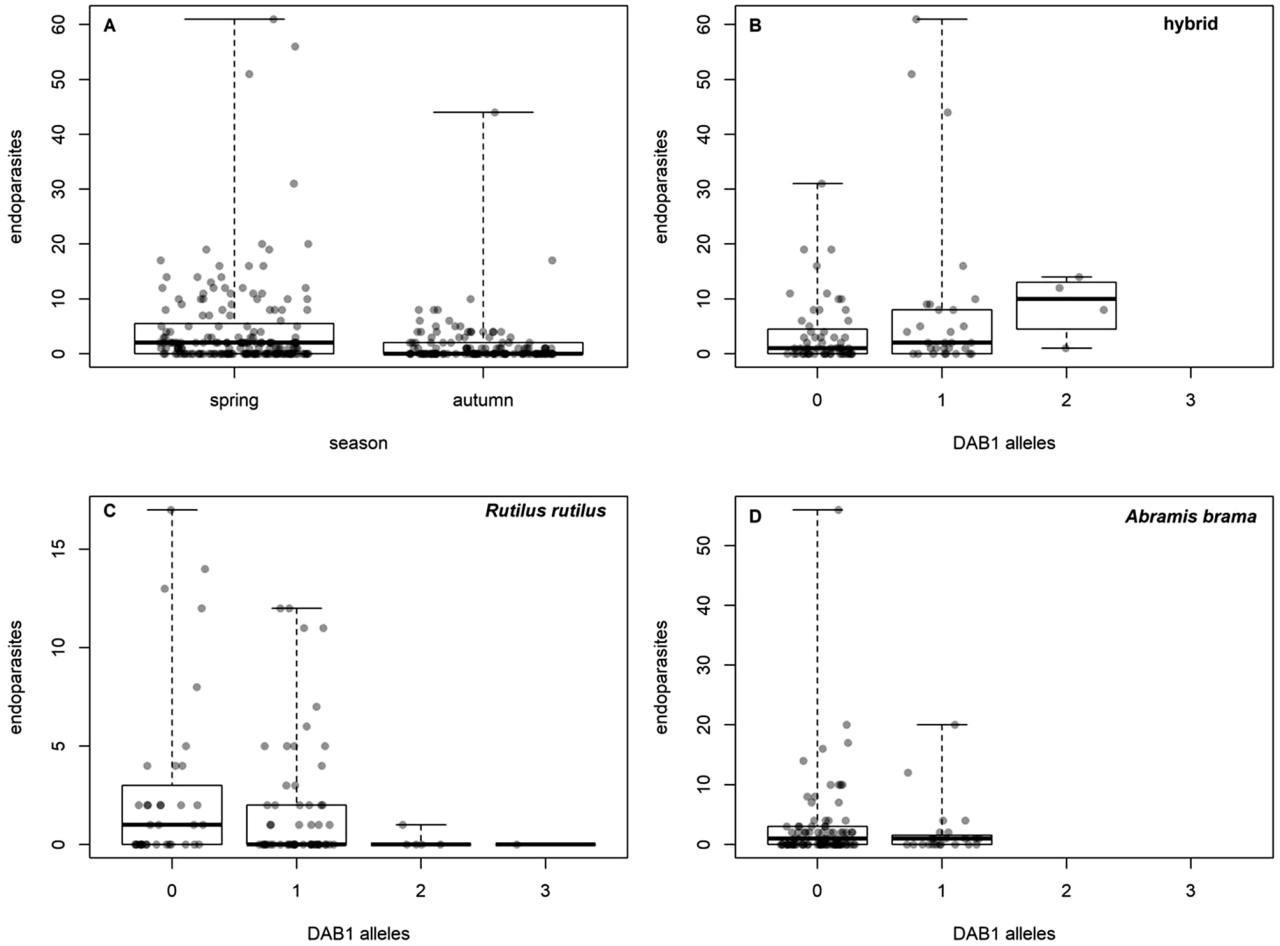
Effect of season on endoparasite abundance (**A**) and effect of the number of *DAB1* alleles on endoparasite abudance in hybrids (**B**), *R. rutilus* (**C**) and *A. brama* (**D**).

The effects of numbers of *DAB1* or *DAB3* alleles on parasites were rare, relatively weak, and inconsistent (Table 4, Supplement S6). In the case of endoparasites, a significant interaction between fish group and number of *DAB1* alleles resulted from the increase in abundance with the number of *DAB1* alleles in hybrids, with no such trend occurring in roach or bream (Fig. 6). In the case of *Gyrodactylus*, there was a significant effect of the interaction between species and number of *DAB3* alleles, with *Gyrodactylus* prevalence decreasing with the number of *DAB3* alleles in roach; no such trend was observed in bream or hybrids. The prevalence of Nematoda was affected by fish group (bream was most highly infected, while no infection was found in roach) and a decrease in the number of *DAB1* alleles. However, the total prevalence of Nematoda infection was very low (see Table 3).

When analyses were performed separately for bream, roach, and hybrids in order to examine the effects of the most common *DAB* alleles on parasite load, GLMM revealed a strong effect of season on parasite load but only limited effects of *DAB* alleles (Table 5). In roach, the presence of the Ruru-DAB3*06 allele had a positive association with *Argulus foliaceus* prevalence and a negative association with *D. similis* abundance, while the presence of the Ruru-DAB1*01 allele had a negative association with *D. caballeroi* abundance and a positive association with *D. micracanthus* prevalence. In bream, the presence of the Arbr-DAB3*03 allele had a negative association with *Ergasilus sieboldi* abundance and a positive association with *G. vimbi* prevalence. No other significant associations between *DAB* alleles and parasite abundance or prevalence were observed (Table 5).

**Table 5:** Predictors remaining in final models originating by backward stepwise regression from GLMMs detecting effects of season and most common *DAB* alleles on attributes describing parasitofauna of roach, common bream and their hybrids (richness, abundance or, in case of prevalence < 30%, prevalence was used instead of abundance).

Response variable	Roach				Hybrids				Common bream			
	c	Term	P	AICc	c	Term	P	AICc	c	Term	P	AICc
Total richness	r^1^	Season	< 0.001	24.3	r^1^	Season	< 0.001	10.8	r^1^	Season	0.001	8.5
*Dactylogyrus* spp.	r^1^	Season	0.003	6.8	r^1^	None			r^1^	None		
Total abundance	a^2^	Season	< 0.001	14.1	a^2^	Season	< 0.001	9.3	a^2^	Season	< 0.001	57.5
Ectoparasite abundance	a^2^	Season	< 0.001	11.5	a^2^	Season	0.001	8.5	a^2^	Season	< 0.001	56.3
Endoparasite abundance	a^2^	Season	< 0.001	20.2	a^2^	Season	0.033	2.3	a^2^	None		
*Diplostomum* spp.	a^2^	None			a^2^	None			a^2^	None		
*Ergasilus sieboldi*	p^4^	None			a^2^	None			a^3^	DAB3*03	0.027	2.8
*Argulus foliaceus*	p^4^	**Season**	0.002	7.7	a^2^	Season	< 0.001	10.9	a^3^	Season	< 0.001	35.5
		DAB3*06	0.010	4.5								
*Tylodelphys clavata*	p^4^	Season	0.035	2.3	a^2^	None			–	–	–	–
*Neoechinorhynchus rutili*	p^4^	Season	< 0.001	21.5	–	–	–	–	–	–	–	–
Caryophyllaeidae spp.	p^4^	Season	< 0.001	20.9	p^4^	None			p^4^	Season	0.017	3.5
*D. crucifer*	a^2^	Season	0.012	4.2	a^2^	Season	0.037	2.1	–	–	–	–
*D. suecicus*	a^2^	Season	0.029	2.6	a^2^	Season	0.003	6.5	–	–	–	–
*D. nanus*	a^2^	None			a^2^	None			–	–	–	–
*D. caballeroi*	a^2^	**Season**	< 0.001	49.5	p^4^	None			–	–	–	–
		DAB1*01	0.011	3.8								
*D. similis*	a^2^	**Season**	< 0.001	50.1	p^4^	Season	< 0.001	9.4	–	–	–	–
		DAB3*06	0.003	6.8								
*D. sphyrna*	p^4^	**Season**	0.030	2.4	p^4^	None			–	–	–	–
*D. micracanthus*	p^4^	DAB1*01	0.001	8.6	p^4^	None			–	–	–	–
*D. rutili*	p^4^	Season	0.28	2.5	–	–	–	–	–	–	–	–
*D. wunderi*	–	–	–	–	–	–	–	–	a^2^	None		
*D. zandti*	–	–	–	–	–	–	–	–	a^2^	None		
*D. auriculatus*	–	–	–	–	p^4^	Season	0.004	6.0	a^2^	Season	< 0.001	852.0
*G. vimbi*	p^4^	Season	0.008	4.9	–	–	–	–	p^4^	**Season**	< 0.001	17.9
										DAB3*03	0.006	5.5
*G. carassii*	p^4^	None			–	–	–	–	–	–	–	–
*G. elegans*	–	–	–	–	p^4^	Season	0.011	4.3	p^4^	Season	< 0.001	11.4

Terms of models where number of alleles played a role are in bold. c = characteristic studied: r = richness, a = abundance, p = prevalence; distribution as specified in the model: ^1^Poisson, ^2^negative binomial, ^3^Poisson with observation level random effects, ^4^Bernoulli. Full model predictors for roach: season + Ruru-DAB1*01 + Ruru-DAB3*06 + Ruru-DAB3*12 + Ruru-DAB3*13. Full model predictors for common bream: season + Arbr-DAB1*02 + Abbr-DAB3*01 + Abbr-DAB3*02 + Arbr-DAB3*03 + Arbr-DAB3*24. Full model predictors for hybrids: season + *Ruru*-DAB1*01 + Abbr-DAB3*02 + Ruru-DAB3*06 + Arbr-DAB3*24. Abbreviations of *DAB* alleles are applied in table.

When tested for the spring season only (Supplement S7), the GLMM test confirmed the signficance of Ruru-DAB3*06 effects on *A. foliaceus* and *D. similis* and a Ruru-DAB1*01 effect on *D. caballeroi* in roach, with other associations becoming non-significant. In contrast, total parasite abundance, ectoparasite abundance, and the abundance of *A. foliaceus* and *E. sieboldi* (the two crustaceans comprising 49% of spring ectoparasites and 37% of all spring parasites) became significantly positively associated with the presence of the Ruru-DAB1*01 allele in hybrids when tested for spring samples only.

When analyses were performed separately for bream, roach, and hybrids in order to examine the effects of the most common MHC supertypes on parasite load, GLMM revealed a strong effect of season on parasite load but only limited effects of MHC supertypes (Table 6). In roach, the presence of supertype E had a positive association with *A. foliaceus* prevalence and negative association with *D. similis* abundance. In addition, the presence of supertype A had positive association with *D. caballeroi*, *D. similis* and *D. micracanthus*. Finally, the presence of supertype C had negative association with *Tylodelphys clavata.* In hybrids, supertype A was associated with total parasite abundance, ectoparasite abundance, endoparasite abundance and abundance of *T. clavata*. In contrast, supertype E was negatively associated with *Diplostomum* spp. and *D. micracanthus,* but positively associated with *G. elegans* prevalence. In bream, supertype E was negatively associated with *E. sieboldi* prevalence and positively associated with *G. vimbi* prevalence. The presence of supertype A was negatively associated with *D. zandti* abundance. When tested for the spring season only (Supplement S8), very similar associations between MHC supertypes and parasite load were found. Four associations reported between MHC supertypes and parasite load for whole sample were also found using spring season only. Moreover, positive association between supertype C and *A. foliaceus* abundance, and negative association between supertype E and *D. micracanthus* abundance were found. In hybrids, five out of seven associations were revealed by both analyses i.e. using whole sample and using spring only. In bream, only the association between supertype E and *G. vimbi* prevalence was the same in both analyses. Using spring sample, two supertypes—D and E were positively associated with *D. zandti* abundance.

**Table 6:** Predictors remaining in final models originating by backward stepwise regression from GLMMs detecting effects of season and most common *DAB* allele supertypes on attributes describing parasitofauna of roach, common bream and their hybrids (richness, abundance or, in case of prevalence < 30%, prevalence was used instead of abundance).

Response variable	roach				hybrids				common bream			
	c	Term	P	AICc	c	Term	P	AICc	c	Term	P	AICc
Total richness	r^1^	Season	< 0.001	24.3	r^1^	Season	< 0.001	10.8	r^1^	Season	0.001	8.5
*Dactylogyrus* spp.	r^1^	Season	0.003	6.8	r^1^	None			r^1^	None		
Total abundance	a^2^	Season	< 0.001	14.1	a^2^	Season	< 0.001	9.8	a^2^	Season	< 0.001	57.5
						**A p**	0.010	4.3				
Ectoparasite abundance	a^2^	Season	< 0.001	11.5	a^2^	Season	0.001	8.5	a^2^	Season	< 0.001	56.3
						**A p**	0.040	2.0				
Endoparasite abundance	a^2^	Season	< 0.001	20.2	a^2^	Season	0.025	2.8	a^2^	None		
						**A p**	0.018	3.3				
*Diplostomum* spp.	a^2^	None			a^2^	**E n**	0.015	3.7	a^2^	None		
*Ergasilus sieboldi*	p^4^	None			a^2^	None			a^3^	**E n**	0.022	3.1
*Argulus foliaceus*	p^4^	Season	< 0.001	9.0	a^2^	Season	< 0.001	11.4	a^3^	Season	< 0.001	35.5
		**E p**	0.015	3.8		**A p**	0.018	3.3				
*Tylodelphys clavata*	p^4^	**C n**	0.033	2.4	a^2^	None			–	–	–	–
*Neoechinorhynchus rutili*	p^4^	Season	< 0.001	21.5	–	–	–	–	–	–	–	–
Caryophyllaeidae spp.	p^4^	Season	< 0.001	20.9	p^4^	None			p^4^	Season	0.017	3.5
*D. crucifer*	a^2^	Season	0.012	4.2	a^2^	Season	0.037	2.1	–	–	–	–
*D. suecicus*	a^2^	Season	0.029	2.6	a^2^	Season	0.003	6.5	–	–	–	–
*D. nanus*	a^2^	None			a^2^	None			–	–	–	–
*D. caballeroi*	a^2^	Season	< 0.001	50.2	p^4^	None			–	–	–	–
		**A p**	0.016	3.5								
*D. similis*	a^2^	Season	< 0.001	49.1	p^4^	Season	< 0.001	9.4	–	–	–	–
		**A p**	0.035	2.2								
		**E n**	0.024	2.8								
*D. sphyrna*	p^4^	Season	0.030	2.4	p^4^	None			–	–	–	–
*D. micracanthus*	p^4^	**A p**	0.003	6.3	p^4^	**E n**	0.013	4.0	–	–	–	–
*D. rutili*	p^4^	Season	0.028	2.5	–	–	–	–	–	–	–	–
*D. wunderi*	–	–	–	–	–	–	–	–	a^2^	None		
*D. zandti*	–	–	–	–	–	–	–	–	a^2^	**A n**	0.020	3.2
*D. auriculatus*	–	–	–	–	p^4^	Season	0.004	6.0	a^2^	Season	< 0.001	852.0
*G. vimbi*	p^4^	Season	0.008	4.9	–	–	–	–	p^4^	Season	< 0.001	18.7
										**E p**	0.004	6.2
*G. carassii*	p^4^	None			–	–	–	–	–	–	–	–
*G. elegans*	–	–	–	–	p^4^	Season	0.030	2.5	p^4^	Season	< 0.001	11.4
						**E p**	0.005	5.6				

MHC supertypes that were a significant part of final models are in bold (p and n stands for positive and negative relationship with the response variable). c = characteristic studied: r = richness, a = abundance, p = prevalence; distribution as specified in the model: ^1^Poisson, ^2^negative binomial, ^3^Poisson with observation level random effects, ^4^Bernoulli. Full model predictors for roach: season + *A* + *C* + *E*. Full model predictors for common bream and hybrids: season + *A* + *C* + *D* + *E.*

## Discussion

Hybridization is a common phenomenon in fish^9,40^; however, prior to this study, the variability in functional immune genes in naturally distributed fish hybrids coexisting in the same natural habitats with their parental species was only rarely investigated^26^. Most analyses of functional immune genes come from experimental studies, e.g. field mesocosm experiments using *Gasterosteus aculeatus*^41,42^. The hybridization between evolutionarily divergent bream and roach is one of the best documented cases of hybridization in wild living cyprinoids. In our study, we hypothesized that MHC diversity in F1 hybrids may reflect the hybrid advantage of being less parasitized when compared to their parental species due to the heterosis effect. A lower level of parasite infection in F1 hybrids of bream and roach was documented^35^. As MHC molecules play a central role in parasite recognition and elimination, following previous studies documenting potential associations between MHC diversity or MHC genotype and metazoan parasites in fish^39,43,44^, we investigated the extent of similarities in MHC profiles between hybrids and each of the parental species. In addition, we examined the potential associations between the most common MHC alleles or MHC supertypes and parasite load.

In our study, we are dealing with the bidirectional hybridization of evolutionarily divergent cyprinoids exhibiting divergent specific metazoan parasitofauna (with a large proportion of host-specific gill and skin monogeneans). Our finding that hybridization of bream and roach was almost perfectly bidirectional is surprising, given previous research in other European countries suggesting that territorial behavior of bream males reduces the opportunity for mating with roach females^32,45^.

We showed that bream and roach tended to express highly divergent MHC profiles, and only a few alleles were shared between the two cyprinoid species as a result of the trans-species evolution widely documented in MHC of fish^10,46,47^. Phylogenetic reconstruction of MHC alleles seems to indicate that genetic introgression may operate in the hybridizing system studied. The previous study in cyprinoids indicated that MHC diversity in populations of hybridzing species is shaped by both trans-species and genetic introgression^26^. However, Wegner and Eizaguirre^24^ highlighted that it is very difficult to disentangle the underlying signatures of trans-species polymorphism from recent introgression and proposed to investigate recombination rates in the chromosomes carrying the MHC genes. According to the allelic profile of MHC genes, population variability was substantially different in bream and roach in the investigated water reservoir. The origin of this variability is unknown as we have no data on the possible establishment of these two cyprinoid species in the Hamry reservoir by human activity or by their own potential invasion of this artificial reservoir from the surrounding rivers. During our investigations, bream was more abundant than roach. The higher MHC polymorphism reported in roach could potentially indicate the multiple origin of roach entering into the reservoir and/or different times of colonization of two species (more recent colonization by bream). To resolve the origin of fish populations, surrounding fish populations connected with the Hamry reservoir should be studied using multiple genetic markers; however, there are no data concerning the potential sources of fish introduction by human activity in the locality of interest. Our former hypothesis may be supported by the interpopulation study based on the MHC study of native *Parachondrostoma toxostoma* and invasive *Chondrostoma nasus* in their hybrid zones in Southern France^26^, where higher polymorphism at population level was reported in native (i.e. more established) species than in the invasive one (i.e. natural colonizer). In contrast to the study by Šimková et al*.*^26^, we found that higher MHC polymorphism in roach is associated with a higher number of roach-specific alleles (more than twice the number in roach when compared to bream).

For each of the cyprinoid species investigated in the present study, several specific alleles were reported at a high frequency and these specific alleles were also reported at high frequencies in hybrids of the F1 generation. Previously, the data on metazoan parasites in these naturally co-occurring bream, roach, and F1 hybrids were analyzed, and the presence of all parental species-specific ectoparasites (mainly gill and fin monogeneans) in hybrids was demonstrated^35^ (shown also in Table 3); however, parental species-specific parasites reached lower levels of infection in hybrids compared to their infection levels in associated bream or roach hosts. This may indicate that the presence of specific MHC alleles in hybrid genomes determines the presence of host-specific parasites or, alternatively, if MHC plays a role in host-parasite coadaptation, that a lack of co-adapted MHC alleles determines a low degree of hybrid susceptibility to host-specific parasites of both species. Previous studies have suggested that direct hybridization in cyprinoids (resulting in the F1 generation of hybrids) likely precludes high intensities of parental species-specific parasites in non-coadapted host genomes^11,26,35^, which may potentially represent one of the advantages of hybrid heterosis for the F1 generation. However, this hypothesis should be carefully reinvestigated using hybrid genomes with expected higher genetic incompatibilities associated with hybrid breakdown, i.e. backcrossed and F2 generations of hybrids.

In the present study, some hybrid-specific MHC allelic variants were observed, a pattern which was previously documented also for hybrids of *Parachondrostoma toxostoma* and *Chondrostoma nasus*^26^. The majority of these hybrid-specific alleles originated from roach, as indicated by phylogenetic reconstruction, a species with higher MHC diversity at the population and individual levels. In accordance with Šimková et al*.*^26^, we found that the MHC variability in hybrids was intermediate between parental species. It was hypothesized that the hybridization of highly divergent species with a likely divergent MHC repertoire increased the number of MHC alleles in hybrids^22^. A high number of MHC variants then leads to the recognition of a high number of foreign antigens by T-cells but is also responsible for the elimination of self-derived peptides (shown for MHC I in the bank vole (*Myodes glareolus*)^48^). In accordance with this hypothesis, high parasite infection should be observed in hybrids, which is not, however, the case of cyprinoid hybrids of the F1 generation^11,35,49^.

In this study, we demonstrated some maternal effect on the MHC expression profile of hybrids. The expression of parental specific MHC alleles was slightly biased in accordance with the maternal origin of hybrids, i.e. hybrids with roach maternal origin expressed a higher number of *DAB3* alleles recognized in roach. The most common allele of roach, Ruru-DAB1*01, and the most common allele of bream, Abbr-DAB3*02, were present in F1 hybrids with roach or bream in the maternal position. However, only hybrids with bream in the maternal position expressed one of the common alleles of bream, i.e. Arbr-DAB1*02. Hybrids with roach maternal origin expressed one of the specific alleles of roach, i.e. Ruru-DAB3*06, at a higher frequency than hybrids with bream maternal origin. The expression of some MHC alleles specific to one or the other parental species may be potentially related to the different levels of infection by some metazoan parasite species (digeneans and crustaceans) previously shown for bream and roach^35^.

We showed that hybrids express an intermediate number of positively selected sites (potential reflecting optimal MHC), and finally, we revealed that hybrids carried lower levels of metazoan parasite infection when compared to parental species (see also^35^). This finding could be interpreted as representing an advantage for hybrids over their parental species. However, it should be also taken into account that hybrids less infected by parental species-specific parasites (monogeneans representing the dominant part of parasite communities) reached higher levels of infection by generalist parasites (especially by digeneans and partially by crustaceans).

The spectrum of *DAB* alleles expressed in hybrids included approximately 18% of the alleles that were not shared with bream and roach specimens. Crossover between non-sister chromatids during meiosis may potentially represent the molecular mechanism leading to generation of new MHC variants in hybrids. Previously, Šimková et al*.*^26^ suggested that low sample size may generate hybrid-specific alleles in the MHC data set. However, it does not seem to be a case in our study as about 100 specimens for each of cyprinoid species and their hybrids were investigated (alternatively even more higher sample size is necessary when investigating MHC genes with high level of polymorphism). Mathematical simulations suggest that frequency-dependent selection resulting from Red Queen dynamics and promoting the advantage conferred by novel alleles may be considered a more important mechanism driving MHC diversity than the mechanism of heterozygote advantage^50,51^. This was evidenced in the study of guppies (*Poecilia reticulata*) experimentally infected by the common monogenean ectoparasite *Gyrodactylus turnbulli*^52^. In the study of guppies, it was shown that host specimens carrying new MHC variants experienced a 35–37% reduction in the intensity of parasite infection, but that the number of MHC variants carried by an individual was not a significant predictor of parasite load^52^. However, the populations of three-spined stickleback exposed to a wider range of parasites tended to be more diverse in MHC IIB genes, and it was proposed that high MHC diversity in wild species is more likely a result of multiple host gene and parasite coevolution^21^. Our results agree with Philips et al.^52^ as we did not identify clear evidence supporting that the number of MHC alleles is a predictor of parasite load, i.e. parasite species richness and especially the abundance of common ectoparasite groups were not associated with the number of MHC alleles. However, it should be also taken into account that the links between MHC and parasites may be underpowered in statistical analyses as previously suggested by Gaigher et al*.*^53^ when studying a link between MHC variation and immunocompetence in wild populations.

Our study does not indicate that *DAB* genes represent the functional immune genes involved in the long-termed coadaptation of host-specific monogeneans and their associated hosts, at least when considering the presence of the most commonly expressed MHC alleles or MHC supertypes (grouping MHC alleles based on functional similarities). We performed COIA analyses using different data sets. No associations between parasites and MHC supertypes were evidenced. Using parasite group data and MHC allele data, we found no significant correlation. However, presence of Abbr-DAB3*10 was weakly associated with Digenea in bream and hybrids, which are both more susceptible to digenean infection when compared to roach. Another potential association was found between one of the most common alleles Abbr-DAB3*1 reported in bream and hybrids and infection by Crustacea (Supplement S3). Even though the correlation between parasite species data and MHC allele data was revealed by COIA in our study, the output of this analysis only clearly demonstrated the strong differentiation of two divergent cyprinoid species based on their specific MHC allelic profiles and the presence of host-specific parasites. Primarily, we can interpret the associations between MHC genes and parasites by two non-mutually exclusive hypotheses: (1) MHC genes may represent the candidate immune genes reflecting long-term host-parasite coadaptation^12,54^—in such a case the species-specific MHC alleles should be associated with the presence of host-specific parasite species, and/or (2) MHC is under local adaptation, i.e. the expression of the most common MHC alleles reflects the increasing local intensity of infection by the most common parasite as a result of negative frequency-dependent selection between the most common host and parasite genotypes^55^, as shown for fish^49^.

However, using a univariate approach based on abundance or prevalence parasite data, only a few associations were revealed between the most common *DAB* alleles and the most common parasite groups or parasite species for roach or bream, although it was possible that these associations were generated randomly because of the high number of statistical tests performed. More specifically, Ruru-DAB1*01 and Ruru-DAB3*06 alleles in roach may be involved in associations with more abundant species (two roach-specific *Dactylogyrus* species and generalist *Argulus foliaceus*), and the presence of Arbr-DAB3*03 may be related to the generalist *Ergasilus sieboldi* infection level in bream. Even though not statistically supported, the presence of Ruru-DAB1*01 and Ruru-DAB3*06 alleles also tended to be related to ectoparasite load in hybrids. This finding may potentially explain the higher proportion of roach-specific parasites in hybrids shown by Krasnovyd et al*.*^35^. By applying a univariate approach with MHC supertype data, the associations observed between MHC supertypes and parasite load were more stable when comparing the outputs from analyses using whole data set or spring only (spring is associated with higher ectoparasite and endoparasite abundance in bream, roach and hybrids when compared to autumn, for details see Krasnovyd et al*.*^35^). The most interesting associations seem to be those reported between MHC supertypes and host-specific *Dactylogyrus* with moderate infection level in roach, and between MHC supertypes and a host-specific and abundant *Dactylogyrus* (*D. zandti*) in bream. In contrast, our study seems to suggest that the presence of supertype A being positively associated with total parasite load may potentially represent some disadvantage for hybrids. Based on the evidence that MHC alleles were not involved strictly in the associations of host-specific parasites and that the most frequent alleles only acted weakly in determining ectoparasite load (also including non-specific parasites), our findings seem to indicate that frequency-dependent selection may partially play a role in the host-parasite associations of the studied fish system. Following limited evidence supporting the associations between functional characteristics of MHC genes (reflected by MHC supertypes) and parasite load, our present study cannot support the hypothesis that MHC genes are involved in the system of long-term host-parasite coadaptation which has been predicted between host-specific parasites and their associated hosts. However, we highligt the need for experimental studies with single species infection under control conditions to investigate the potential associaton betwen specific MHC supertypes and host-specific parasites; such studies may more rigorously infer the role of MHC genes in host-parasite coadaptation.

High MHC polymorphism is most pronounced in the peptide-binding regions. As the PBR of fish have not yet been determined, their putative positions may be determined on the basis of the maximum likelihood approach of codon-based models (the M8 model most rigorously indicating the action of positive selection at amino-acid sites) and then revealed by PSS (applied to fish, e.g.^26,49^). These specific amino-acid sites are in direct contact with the bound peptides that mediate the recognition of foreign antigens derived from pathogens and parasites. We identified a wide range of identical PSS in both divergent cyprinoid species and hybrids, which seems to suggest the role of these sites at the MHC molecule for potentially recognizing the parasite group or phylogenetically related (congeneric) parasite species. Previous studies showed a high number of PSS in the species or form which was under stronger parasite selection. More specifically, a slighly higher number of PSS were found in invasive *C. nasus* infected by *Chondrostoma*-specific parasites^26^ then in native *P. toxostoma* non-infected by these monogeneans. The most obvious difference was found in the number of PSS between asexual and sexual forms of gibel carp (*Carassius gibelio*), with a higher number of PSS and a higher intensity of infection of host-specific *Dactylogyrus* in the asexual form than in the sexual form^49^. According to the M8 model, we detected a slightly higher number of PSS in bream, which also reached a higher level of parasite infection (especially for the most common ectoparasite groups—monogeneans and crustaceans) when compared to roach. Interestingly, we found that one PSS was shared only between bream and hybrids and that another PSS was shared only between roach and hybrids. We propose that this specific sharing of peptide-binding regions of MHC genes between hybrids and each of the parental species may determine the low susceptibility of hybrids to acquiring parental-species-specific parasites. In addition, species-specific PBR may even reflect potential coevolutionary interactions between host-specific parasites and hosts carrying these amino-acid sites. However, such a hypothesis could only be seriouly examined on the basis of the structure of MHC molecules in two divergent fish species and the interactions between molecular components of both MHC genes and host-specific parasites.

In conclusion, we identified clear difference in both MHC profile and parasite communities of evolutionarily divergent leuciscid species, i.e. bream and roach expressed many specific MHC alleles and harboured many host-specific parasites (especially representetives of ectoparasitic monogeneans). The intermediate MHC diversity (in term of numbers of *DAB* alleles and peptide-binding sites) in hybrids may be considered as optimal MHC diversity potentially associated with low total parasite load, which is in line with the hybrid advantage hypothesis. Sharing of the most frequent alleles and peptide-binding regions of MHC genes between hybrids and parental species may potentially determine the susceptibility of hybrids to acquire parental-species-specific parasites in low abundance. However, our study did not reveal clear associations between species-specific MHC alleles and host-specific parasites, suggesting that potentially genes other than that of MHC are primarily involved in the coevolutionary associations of hosts and their host-specific parasites.

## Methods

Bream (*Abramis brama*), roach (*Rutilus rutilus*), and their respective hybrids were collected from the Hamry reservoir (49.737N, 15.914 E; the Czech Republic) during spring and autumn of three consecutive years. A fin clip from each specimen was preserved in 96% ethanol for further molecular identification. Fish were identified by morphology, later confirmed with genetic markers (partial mitochondrial cyt *b* gene to determine the maternal origin and 12 microsatellite loci, see^35^). All hybrids were assigned as F1 hybrids^35^. Fish were transported to the laboratory and dissected for metazoan parasites^35^. All fish manipulation was in accordance with Law No. 207/2004 of the collections of Laws of the Czech Republic on the protection, breeding and use of experimental animals. The study was approved by the Animal Care and Use Committee of the Faculty of Science, Masaryk University in Brno (Czech Republic). All methods were carried in accordance with ARRIVE guidelines.

Spleen samples were collected from individual fish and stored in RNAlater stabilization solution (Ambion) at -80 ˚C. RNeasy Mini Kit (Qiagen) and High Capacity RNA-to-cDNA Kit (Thermo Fisher Scientific) were used for RNA isolation and reverse transcription, respectively. The obtained cDNA was subsequently used as a template in PCR reactions in order to amplify the whole exon 2 of MHC class II*B* genes (i.e., *DAB1* and *DAB3* genes) using the primers and protocol applied by Šimková et al*.*^26^. Samples were subjected to MiSeq Illumina sequencing (provided by CEITEC, Brno, Czech Republic). The SESAME program^56^ was used to visualize the obtained next-generation data. Putative true alleles were evaluated as described in Šimková et al*.*^26^. More specifically, to distinguish true alleles from artefacts generated at different stages of MHC genotyping, we followed the procedures of Zagalska-Neubauer et al*.*^57^ as applied by Nadachowska-Brzyska et al*.*^23^. Briefly, we excluded all variants containing indels causing frameshifts or present in one copy in all data sets. We considered all amplicons with sufficient coverage and we calculated the maximum per-amplicon frequency (MPAF) for each sequence variant. Each sequence variant with an MPAF of less than 5% was checked for potential artefacts (i.e. 1 bp substitution or recombination from other sequence variants in a given amplicon). The sequence variants present in ≥ 50% of the amplicon’s reads were not explained by the artefacts and were considered as the true alleles in our study. A large proportion of true alleles were confirmed by two independent PCRs. The genotyping of all specimens for which the singletons were found was repeated twice.

Designation of all *DAB* alleles to *DAB1* and *DAB3* lineages follows Van Erp et al*.*^58^, for the comprehensive review on MHC genes in cyprinoid fish see Šimková^59^. The specific alleles of *A. brama* were termed *Abbr-DAB* alleles and the specific alleles of *R. rutilus* were termed *Ruru-DAB* alleles, following the nomenclature of Klein et al.^60^. The alleles identified in both *A. brama* and *R. rutilus* and their hybrids, as well as the alleles identified solely in hybrids were termed *Arbr-DAB* alleles according to Šimková et al*.*^26^. The terminology *DAB1* alleles and *DAB3* alleles was applied following previous studies on MHC in cyprinoids^26,61–63^. The new *DAB* alleles were deposited in GenBank under Accession numbers from MW737269 to MW737373.

We used Kruskal–Wallis H-test with multiple comparisons to compare the number of *DAB* alleles between *A. brama, R. rutilus*, and their respective hybrids. The MrBayes program^64^ was used to infer a phylogenetic tree based on all detected *DAB* alleles. The resulting tree was constructed using the Metropolis-coupled Markov chain Monte Carlo algorithm. Four concurrent chains (one cold and three heated) ran for 10^7^ generations, sampling trees every 10^2^ generations. The first 30% of trees were discarded as a relative burn-in period after checking that the standard deviation split frequency fell below 0.01. The corrected Akaike information criterion was used to calculate the most accurate substitution model in jModelTest^65^.

To detect positive selection on a site-by-site basis, a maximum likelihood approach in the CODEML program implemented in PAML 4.3 was used^66^. Whole data set i.e. *DAB1* and *DAB3* alleles were used for the analyses as previously applied in the study of cyprinoid fish and their hybrids^26^. Different codon-based models incorporating selection (M3, M2a and M8) and not incorporating selection (M0, M1a and M7) were used to test for the presence of sites under selection following Yang^66^. The Bayes empirical Bayes method (BEB) was used to calculate the posterior probabilities for site classes and to identify sites under selection (the posterior means of ω for positively selected sites (PSS) are > 1). BEB is implemented under models M2a and M8.

MHC alleles were clustered into MHC supertypes using the method described by Phillips et al*.*^52^ (see Supplement S9 for detailed information). Based on 24 amino acid sequences identified as being under selective pressure, K-means clustering was used to cluster alleles into supertypes, identifying 8 clusters as optimal *k* based on change in the Bayesian Information Criterion. Discriminant analysis of principal components was then used to identify the probability of an allele being in a particular supertype and assign their modal supertype cluster. Alleles that were not consistent in their supertype clustering were removed from downstream analysis (n = 13, 12%).

All fish specimens were investigated for the presence of metazoan parasites^35^. Potential associations between MHC alleles and metazoan parasites were examined using a multivariate approach. Co-inertia analysis (COIA^67,68^) was applied to investigate the relationships (1) between the MHC genetic matrix including the presence/absence of 14 *DAB* alleles (observed ≥ 5 individuals) and the parasite matrix including the abundance of 25 metazoan parasite species, (2) between the MHC genetic matrix including the presence/absence of 14 *DAB* alleles and metazoan parasite groups (Monogenea, Crustacea, Digenea, Acanthocephala and Nematoda), and (3) between the MHC genetic matrix including the presence/absence of MHC supertypes (8 MHC supertypes) and metazoan parasite groups. From the parasite data set, species with a prevalence ≤ 2% in roach or bream were not included in the analyses. COIA analysis was performed both (i) separately for bream, roach and hybrid individuals and (ii) on a dataset including all three fish groups. The first step of COIA involved the separate analyses of each matrix, i.e., the analysis of the MHC genetic matrix by correspondence analysis (CA), and the analysis of the parasitological matrix by principal component analysis (PCA), using the row weights of the CA. In the second step, the two ordinations were combined in COIA to explore the co-structure between the parasite and the genetic variables. A possible link between the two tables was analyzed by performing pairwise Monte-Carlo permutation tests on the value of the RV coefficient using 999 random permutations. The association between the two matrices was estimated from the COIA map by comparing the distribution of genetic and parasite variables. Usually, when interpreting graphical outputs of COIA analysis, variables located in a given direction relative to the origin are considered to be positively associated, and variables located in the opposite direction are considered to be negatively associated^44,69,70^.

In order to describe univariate patterns in effects of *DAB* alleles on parasite abundance and richness, generalized linear mixed models (GLMM) were implemented in several model series. Sampling year was included as a random factor in all models. First, we tested for the effect of the number of *DAB* alleles on parasites. For each response variable, two full models were constructed: the response variable was modelled to be dependent a) on season (spring versus autumn), fish group (bream, roach, hybrid), number of *DAB* alleles (DAB_t, henceforth) and DAB_t: fish group interaction and b) on season, fish group, number of *DAB1* alleles (DAB1_t, henceforth), number of *DAB3* alleles (DAB3_t, henceforth), and DAB1_t: fish group and DAB3_t: fish group interactions. Due to highly species-specific fish-parasite associations, it was not possible to investigate the effect of these predictors on the species level and we used higher taxonomical and functional groups instead. The following set of response variables was included in these models: parasite richness; *Dactylogyrus* spp. richness; parasite abundance; and the abundance or prevalence of ectoparasites, endoparasites, Monogenea, Digenea, Crustacea, Acanthocephala, Nematoda, Cestoda and the most common genera of Monogenea: *Dactylogyrus* and *Gyrodactylus*. Primarily, we aimed to model taxa abundance, but in less frequent taxonomical groups (prevalence < 30%), the abundance models could be affected by zero inflation; therefore, we decided to model their prevalence instead.

A corresponding error structure, reflecting the distribution of each response variable and residual structure, was attributed to each model: we constructed Poisson models for richness, negative binomial models for abundance, Poisson models with observation level random effects for abundance (when negative binomial models produced patterns in residuals that suggested the model does not describe the data properly), and Bernoulli models for prevalence. For each model, a backward stepwise selection procedure was used to select the best (final) model^71^. We used this procedure, as we aimed for a single final interpretation model. Selection was based on a comparison of AICc (i.e. the Akaike information criterion with correction for finite sample sizes) for each pair of nested models. Models with a ΔAICc value of less than 2 were considered of equal fit^72^, with the simpler model preferred in the selection process (the results of log-likelihood tests comparing the two models were used as a supplementary criterion and are presented in the results and tables).

Further, we aimed to test for the association between individual MHC alleles (or MHC supertypes) and parasites—these models were conducted separately for bream, roach and hybrids due to high interspecific differences in *DAB* allele composition. The response variable set for each of these models consisted of parasite richness, *Dactylogyrus* species richness, parasite abundance, the abundances of ectoparasites and endoparasites, and the abundance or prevalence of the most abundant species. The predictor set consisted of season and the presence of every *DAB* allele (or MHC supertype) that occurred in more than 7 individuals of the respective fish group. Alleles or supertypes with lower frequency often caused our models to fail to converge and we considered that including them in the analysis could not provide convincing results. As fish were far more parasitized in spring (by ectoparasites) compared to autumn (see Results), this could potentially have weakened associations observed between individual alleles and parasite species. We, therefore, decided also to conduct a separate analysis for spring samples only to investigate such an effect. All univariate analyses were conducted using R statistical software version 3.5.2^73^, using the *base*^74^, *lme4*^75^ and *MuMIn*^75^ packages for univariate models and *ade4* and *ade4TkGUI*^76^ for multivariate analysis (PCA, CA, COIA, permutation test).

## Supplementary Information

Supplementary Information.

## Data Availability

The sequences of *DAB* alleles obtained during this study were deposited in NCBI GenBank under accession numbers MW737269-MW737373.
