# Supplementary material for: Diversity of MHC IIB genes and parasitism in hybrids of evolutionarily divergent cyprinoid species indicate heterosis advantage
Source: Sci Rep. 2021 Aug 19;11:16860. doi: 10.1038/s41598-021-96205-x (PMC8376869; doi:10.1038/s41598-021-96205-x)
Supplement: Supplementary file 1 — Supplementary Information. [file 41598_2021_96205_MOESM1_ESM.pdf]

Diversity of MHC IIB genes and parasitism in hybrids of evolutionarily divergent cyprinoid species indicate heterosis advantage

Andrea Šimková[1*], Lenka Gettová[1], Kristína Civáňová[1], Mária Seifertová[1], Michal Janáč[2] and Lukáš Vetešník[1, 2]

[1]Department of Botany and Zoology, Faculty of Science, Masaryk University, Kotlářská 2, 611 37 Brno, Czech Republic

[2]Institute of Vertebrate Biology, Academy of Sciences of the Czech Republic, Květná 8, 603 65 Brno, Czech Republic

*corresponding author: simkova@sci.muni.cz

Supplement S1. The frequencies of the most common *DAB* alleles in hybrids with bream in maternal positon (hybrids AB) and with roach in maternal position (hybrids RR). Only the frequencies of the most frequent 9 alleles are shown.

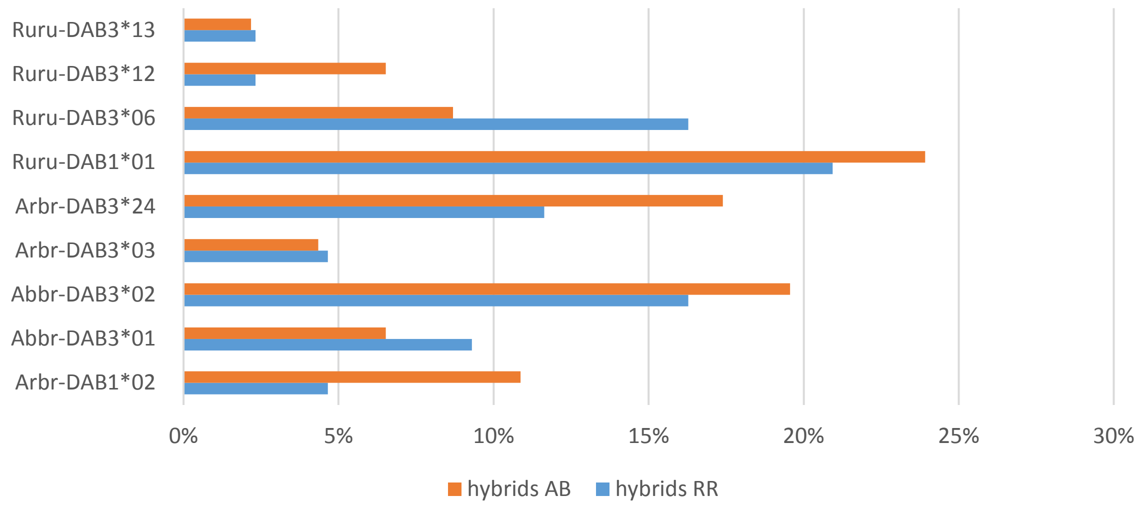

Supplement S2. Log-likelihood values and parameter estimates under random-site models for *A. brama*, *R. rutilus* and their hybrids. ω is the selection parameter; p_n is the proportion of sites that fall into the ω_n site class; p and q are the shape parameters of the β function (for M7 and M8 models). Whole data set (*DAB1* and *DAB3* alleles) were used for analyses.

	Model	Log-likelihood values	Parameters	Number of positive sites (P>99%/P>95%)
A. brama	M0	-2431.78	ω=1.62	
	M1a	-2294.23	p_0=0.865 (p_1=0.135), ω_0=0.051 (ω_1=1)	
	M2a	-2190.43	p_0=0.590, p_1=0.353 (p_2=0.056), ω_0=0.087, ω_1=1.000, ω_2=8.027	23/24
	M3	-2187.42	p_0=0.844, p_1=0.123 (p_2=0.033), ω_0=0.308, ω_1=3.016, ω_2=11.235	
	M7	-2303.94	p=0.022, q=0.141	
	M8	-2190.83	p_0=0.941 (p_1=0.059), p=0.119, q=0.151, ω_s=8.173	26/33
R. rutilus	M0	-4953.78	ω=1.87	
	M1a	-4579.04	p_0=0.925 (p_1=0.075), ω_0=0.072 (ω_1=1)	
	M2a	-4333.42	p_0=0.642, p_1=0.307 (p_2=0.051), ω_0=0.146, ω_1=1.000, ω_2=7.373	23/25
	M3	-4312.04	p_0=0.900, p_1=0.085 (p_2=0.016), ω_0=0.494, ω_1=4.493, ω_2=14.656	
	M7	-4593.28	p=0.020, q=0.134	
	M8	-4335.81	p_0=0.949 (p_1=0.051), p=0.274, q=0.356, ω_s=7.437	25/26
Hybrids	M0	-3863.45	ω=2.14	
	M1a	-3580.09	p_0=0.910 (p_1=0.090), ω_0=0.055 (ω_1=1)	
	M2a	-3366.09	p_0=0.571, p_1=0.381 (p_2=0.049), ω_0=0.064, ω_1=1.000, ω_2=8.743	25/25
	M3	-3361.01	p_0=0.723, p_1=0.231 (p_2=0.045), ω_0=0.205, ω_1=2.039, ω_2=11.800	
	M7	-3582.75	p=0.017, q=0.112	
	M8	-3367.26	p_0=0.951 (p_1=0.049), p=0.093, q=0.118, ω_s=8.777	25/28

Supplement S3. COIA of the MHC alleles and metazoan parasite groups. Color labelling of *DAB* alleles and parasites corresponds to their presence in bream, roach and hybrids.

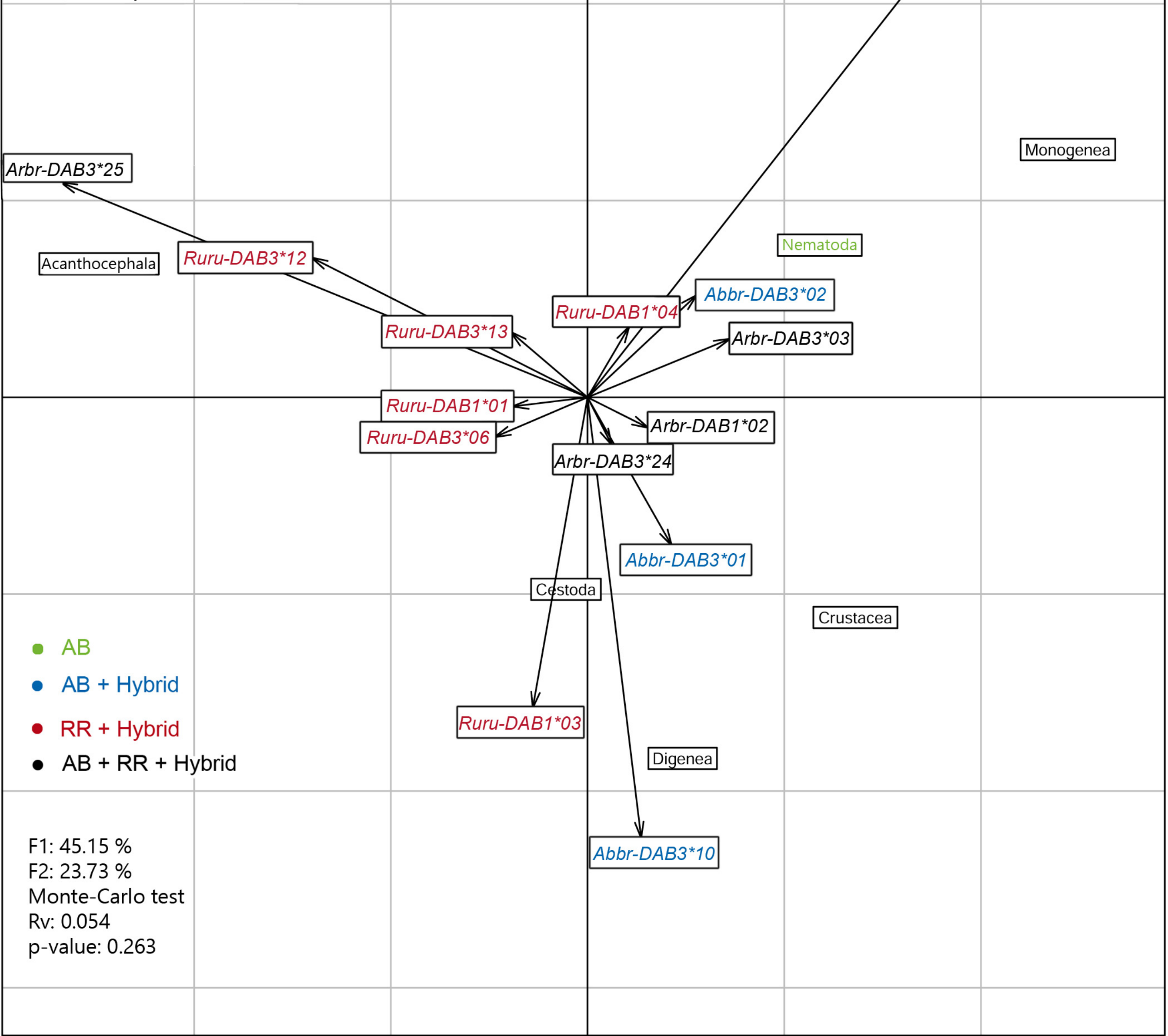

Supplement S4. COIA of the MHC supertypes and metazoan parasite groups.
Color labelling of *DAB* alleles and parasites corresponds to their presence in bream, roach and hybrids.

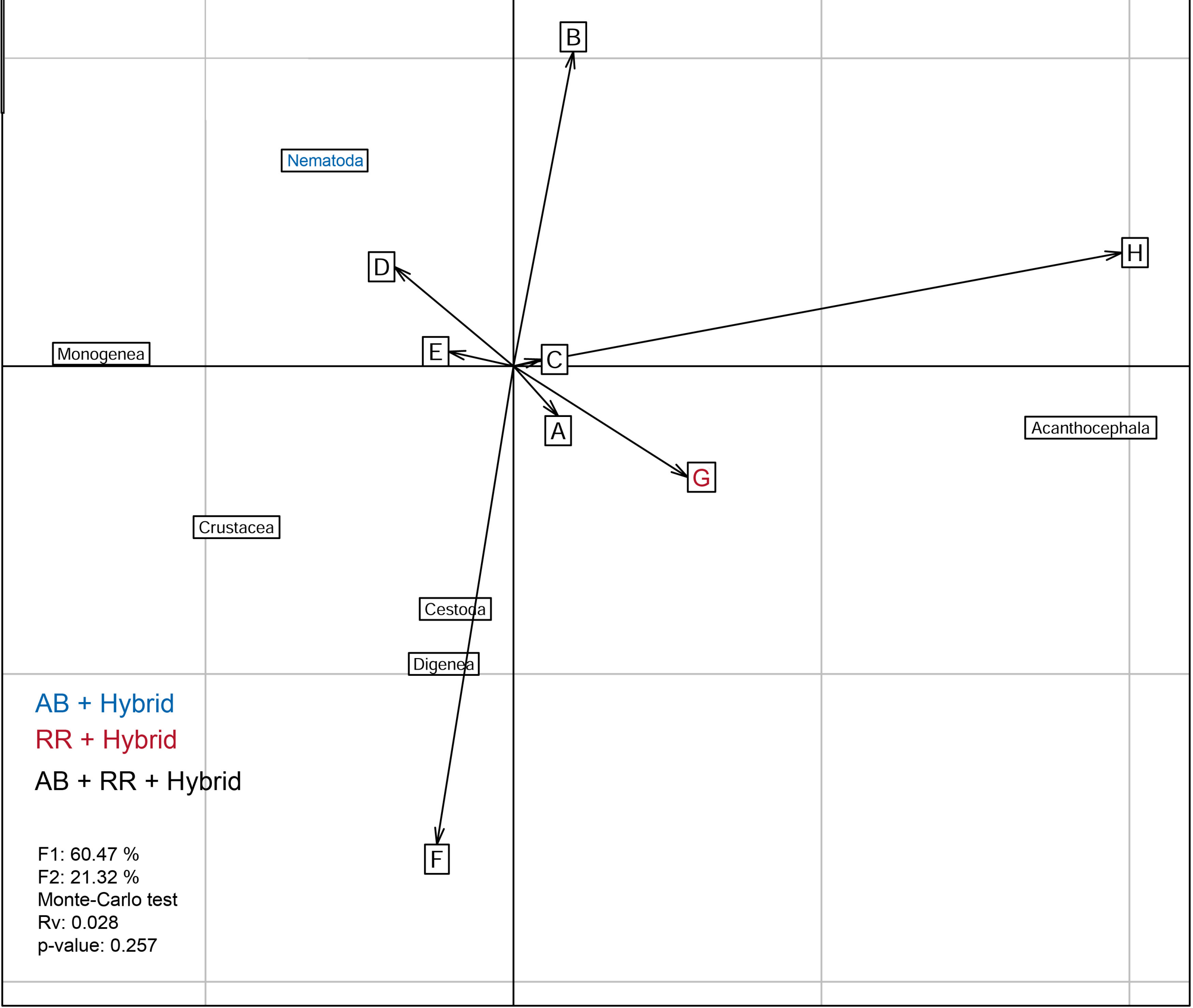

Supplement S5. PCA and COA row scores adjusted according to COIA first axis. Red dots correspond to roach, orange to hybrids, blue to common bream. Note clear separation of common bream and roach individuals on both COA axis (*DAB* alleles) and PCA axis (parasite assemblages). While some of hybrids overlap in MHC genes with roach or common bream individuals, they are always close to the ordination center on both axes (i.e. unimportant for ordination of both MHC genes and parasite assemblages).

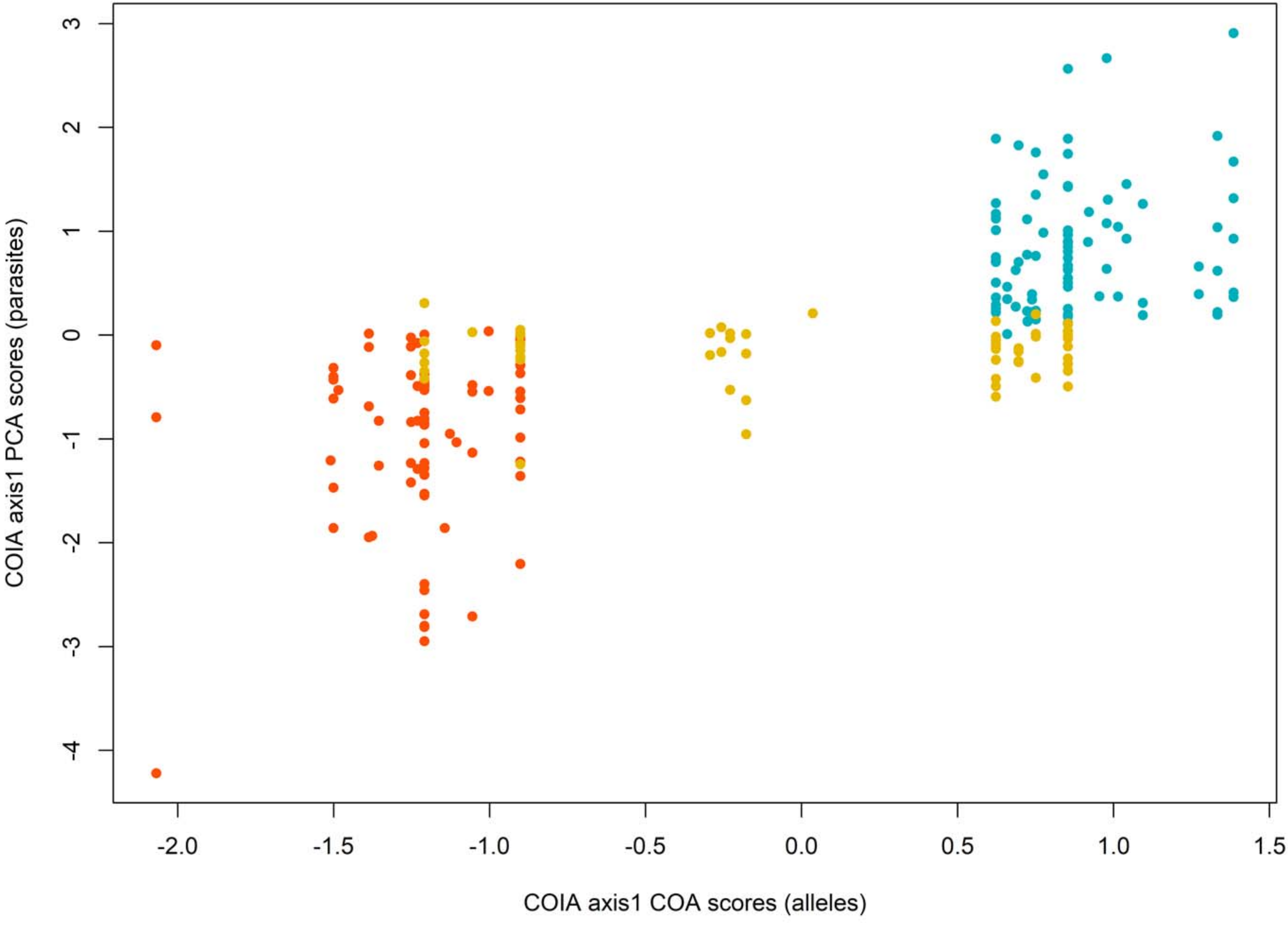

Supplement S6. Final GLMM model terms and their statistics for models predicting effect of season, fish group and number of *DAB1* and *DAB3* alleles. For three-level factor (fish group) differences between each level pair are provided. For season, the effect direction is considered as from spring to autumn (negative values mean decrease in response variable in autumn). Significant effects, their P-value and direction are in bold. NA – statistics were not calculated due to lack of variability (no parasites sampled) in one of the predictors' levels.

response	distribution	predictors	estimate (beta)	SE	z value	P	effect direction	final model AICc
total richness	Poisson	**season**	-0.42	0.05	-7.74	**<0.001**	**-**	1221.8
Dactylogyrus richness	Poisson	**season**	-0.35	0.07	-4.85	**<0.001**	**-**	996.0
		fish group: HY-AB	0.08	0.09	0.92	0.359	+	
		**fish group: HY-RR**	0.47	0.09	5.30	**<0.001**	**+**	
		**fish group: AB-RR**	0.39	0.08	4.55	**<0.001**	**+**	
total abundance	negative binomial	**season**	-1.02	0.10	-10.20	**<0.001**	**-**	2880.7
		**fish group: HY-AB**	1.43	0.12	11.90	**<0.001**	**+**	
		**fish group: HY-RR**	0.96	0.13	7.70	**<0.001**	**+**	
		**fish group: AB-RR**	-0.47	0.13	-3.70	**<0.001**	**-**	
ectoparasite abundance	negative binomial	**season**	-1.01	0.10	-9.77	**<0.001**	**-**	2828.7
		**fish group: HY-AB**	1.65	0.12	13.40	**<0.001**	**+**	
		**fish group: HY-RR**	1.19	0.13	9.18	**<0.001**	**+**	
		**fish group: AB-RR**	-0.46	0.13	-3.64	**<0.001**	**-**	
Monogenea abundance	negative binomial	**season**	-1.02	0.11	-9.06	**<0.001**	**-**	2707.7
		**fish group: HY-AB**	2.24	0.13	16.69	**<0.001**	**+**	
		**fish group: HY-RR**	1.86	0.14	13.13	**<0.001**	**+**	
		**fish group: AB-RR**	-0.38	0.14	-0.27	**0.006**	**-**	
Dactylogyrus abundance	negative binomial	**season**	-0.98	0.11	-8.61	**<0.001**	**-**	2691.7
		**fish group: HY-AB**	2.22	0.14	16.42	**<0.001**	**+**	
		**fish group: HY-RR**	1.86	0.14	13.07	**<0.001**	**+**	
		**fish group: AB-RR**	-0.35	0.14	-2.58	**0.010**	**-**	
Gyrodactylus prevalence	binomial	**season**	-1.61	0.61	-2.65	**<0.001**	**-**	261.3
		fish group: HY-AB	1.08	0.82	1.33	0.182	+	
		**fish group: HY-RR**	1.65	0.76	2.18	**0.029**	**+**	
		fish group: AB-RR	0.57	0.77	0.74	0.459	+	
		DAB3_t HY-AB	-0.03	0.57	-0.06	0.955	-	
		**DAB3_t HY-RR**	-1.41	0.64	-2.20	**0.029**	**-**	
		**DAB3_t AB-RR**	-1.37	0.64	-2.15	**0.032**	**-**	
endoparasite abundance	negative binomial	**season**	-0.98	0.21	-4.62	**<0.001**	**-**	1239.6
		fish group: HY-AB	-0.17	0.30	-0.57	0.565	-	
		fish group: HY-RR	-0.38	0.37	-1.02	0.306	-	
		fish group: AB-RR	-0.21	-0.35	-0.60	0.546	-	
		**DAB1_t HY-AB**	-1.06	0.53	-2.00	**0.046**	**-**	

		**DAB1_t HY-RR**	-1.12	0.48	-2.34	**0.019**	**-**	
		DAB1_t AB-RR	-0.06	0.54	-0.10	0.918	-	
Crustacea abundance	Poisson; observation level random effect	**season**	-0.95	0.19	-4.89	**<0.001**	**-**	1407.7
		fish group: HY-AB	0.08	0.21	0.38	0.706	+	
		**fish group: HY-RR**	-2.31	0.27	-8.55	**<0.001**	**-**	
		**fish group: AB-RR**	-2.39	0.26	-9.11	**<0.001**	**-**	
Digenea abundance	Poisson; observation level random effect	**season**	-0.62	0.27	-2.31	**0.021**	**-**	1074.0
		**fish group: HY-AB**	-1.16	0.31	-3.73	**<0.001**	**-**	
		**fish group: HY-RR**	-1.40	0.33	-4.29	**<0.001**	**-**	
		fish group: AB-RR	-0.25	0.33	-0.75	0.452	-	
Acanthocephala prevalence	binomial	**season**	NA	NA	NA	**NA**	**-**	95.7
		fish group: HY-AB	-0.18	1.03	-0.17	0.863	-	
		**fish group: HY-RR**	1.79	0.82	2.20	**0.028**	**+**	
		**fish group: AB-RR**	1.97	0.88	2.24	**0.025**	**+**	
Nematoda prevalence	binomial	fish group: HY-AB	NA	NA	NA	NA	+	85.0
		fish group: HY-RR	NA	NA	NA	NA	0	
		**fish group: AB-RR**	NA	NA	NA	NA	**-**	
		**DAB1_t**	NA	NA	NA	NA	**-**	
Cestoda prevalence	binomial	season	-2.08	0.54	-3.85	**<0.001**	**-**	216.8

Supplement S7. Predictors remaining in final models originating by backward stepwise regression from GLMMs detecting effect of the most common *DAB* alleles on attributes describing parasitofauna of roach, common bream and their hybrids in spring sampling (richness, abundance or, in case of prevalence ≤ 15 %, prevalence instead of abudance was used). Predictors of models where number of alleles played a role are in bold. c = characteristic studied: r = richness, a = abundance, p = prevalence; distribution as specified in the model: [1] Poisson, [2] negative binomial, [3] Poisson with observation level random effects, [4] Bernoulli. Full model predictors for roach: season + *Ruru-DAB1*01* + *RuRu-DAB3*06* + *Ruru-DAB3*12* + *Ruru-DAB3*13*. Full model predictors for common bream: season + *Arbr-DAB1*02* + *Abbr-DAB3*01* + *Abbr-DAB3*02* + *Arbr-DAB3*03* + *Arbr-DAB3*24*. Full model predictors for hybrids: season + *Ruru-DAB1*01* + *Abbr-DAB3*02* + *Ruru*-DAB3*06 + *Arbr-DAB3*24*. Abbreviations of *DAB* alleles are applied in table.

	roach				hybrids				common bream			
response variable	c	term	P	AICc	c	term	P	AICc	c	term	P	AICc
total richness	r[1]	none			r[1]	none			r[1]	none		
Dactylogyrus spp.	r[1]	none			r[1]	none			r[1]	none		
total abundance	a[2]	none			a[2]	***DAB1*01***	0.011	4.1	a[2]	none		
ectoparasite abundance	a[2]	none			a[2]	***DAB1*01***	0.007	4.9	a[2]	none		
endoparasite abundance	a[2]	none			a[2]	none			a[2]	none		
Diplostomum spp.	a[2]	none			a[2]	none			a[2]	none		
Ergasilus sieboldi	p[4]	none			a[2]	***DAB1*01***	0.007	5.0	a[3]	none		
Argulus foliaceus	p[4]	***DAB3*06***	0.023	2.9	a[2]	***DAB1*01***	0.030	2.3	a[3]	none		
Tylodelphys clavata	p[4]	none			a[2]	none			-	-	-	-
Neoechinorhynchus rutili	p[4]	none			-	-	-	-	-	-	-	-
Caryophyllaeidae spp.	p[4]	none			p[4]	none			p[4]	none		
D. crucifer	a[2]	none			a[2]	none			-	-	-	-
D. suecicus	a[2]	none			a[2]	none			-	-	-	-
D. nanus	a[2]	none			a[2]	none			-	-	-	-
D. caballeroi	a[2]	***DAB1*01***	0.009	4.5	p[4]	none			-	-	-	-
D. similis	a[2]	***DAB3*06***	0.003	6.4	p[4]	none			-	-	-	-
D. sphyrna	p[4]	none			p[4]	none			-	-	-	-
D. micracanthus	p[4]	none			p[4]	none			-	-	-	-
D. rutili	p[4]	none			-	-	-	-	-	-	-	-
D. wunderi	-	-	-	-	-	-	-	-	a[2]	none		
D. zandti	-	-	-	-	-	-	-	-	a[2]	none		
D. auriculatus	-	-	-	-	p[4]	none			a[2]	none		
G. vimbi	p[4]	none			-	-	-	-	p[4]	***DAB3*03***	0.006	5.4
G. carassii	p[4]	none			-	-	-	-	-	-	-	-
G. elegans	-	-	-	-	p[4]	none			p[4]	none		

Supplement S8. Predictors remaining in final models originating by backward stepwise regression from GLMMs detecting effect of the most common *DAB* allele supertypes on attributes describing parasitofauna of roach, common bream and their hybrids in spring sampling (richness and abundance; in case of prevalence ≤ 15 %, prevalence instead of abudance was used). Allele supertypes that were a signifcant part of final models are in bold (p and n stands for positive and negative relationship with the response variable). c = characteristic studied: r = richness, a = abundance, p = prevalence; distribution as specified in the model: [1] Poisson, [2] negative binomial, [3] Poisson with observation level random effects, [4] Bernoulli. Full model predictors for roach: *A* + *C* + *E*. Full model predictors for common bream and hybrids: *A* + *C* + *D* + *E*.

	roach				hybrids				common bream			
response variable	c	term	P	AICc	c	term	P	AICc	c	term	P	AICc
total richness	r^1	none			r^1	none			r^1	none		
Dactylogyrus spp.	r^1	none			r^1	none			r^1	none		
total abundance	a^2	none			a^2	**A p**	0.015	3.6	a^2	none		
ectoparasite abundance	a^2	none			a^2	none			a^2	none		
endoparasite abundance	a^2	none			a^2	none			a^2	none		
Diplostomum spp.	a^2	none			a^2	**E n**	0.031	2.3	a^2	none		
Ergasilus sieboldi	p^4	none			a^2	none			a^3	none		
Argulus foliaceus	p^4	**E p**	0.005	5.6	a^2	**A p**	0.019	3.1	a^3	none		
		**C p**	0.036	2.1								
Tylodelphys clavata	p^4	none			a^2	none			-	-	-	-
Neoechinorhynchus rutili	p^4	none			-	-	-	-	-	-	-	-
Caryophyllaeidae spp.	p^4	none			p^4	none			p^4	none		
D. crucifer	a^2	none			a^2	none			-	-	-	-
D. suecicus	a^2	none			a^2	none			-	-	-	-
D. nanus	a^2	none			a^2	none			-	-	-	-
D. caballeroi	a^2	**A p**	0.013	3.8	p^4	none			-	-	-	-
D. similis	a^2	**E n**	0.003	2.4	p^4	none			-	-	-	-
D. sphyrna	p^4	none			p^4	none			-	-	-	-
D. micracanthus	p^4	**E n**	0.009	4.5	p^4	**E n**	0.013	4.0	-	-	-	-
		**A p**	0.017	3.3								
D. rutili	p^4	none			-	-	-	-	-	-	-	-
D. wunderi	-	-	-	-	-	-	-	-	a^2	none		
D. zandti	-	-	-	-	-	-	-	-	a^2	**D p**	0.009	4.5
										**E p**	0.008	4.7
D. auriculatus	-	-	-	-	p^4	none			a^2	none		
G. vimbi	p^4	none			-	-	-	-	p^4	**E p**	0.004	6.1
G. carassii	p^4	none			-	-	-	-	-	-	-	-
G. elegans	-	-	-	-	p^4	**E p**	0.005	5.4	p^4	none		

Supplement S9. Methodology of MHC supertypes identification including Figure demonstrating change in BIC ($\Delta BIC = BIC_k - BIC_{k+1}$) with additional clusters, and relevant references.

From a 92 amino acid long sequence, 24 positions under selection pressure were chosen for subsequent analysis. Each amino acid was replaced with five Z-scores describing its physicochemical properties according to Sandberg et al. (1998), using *zScales()* function in R package *Peptides* (Osorio et al. 2015). Selecting number of clusters (k) was based on using *find.clusters()* function in R package *adegenet* (Jombart 2008). Following Phillips et al. (2018), 100 repeats of the function was used to determine the optimal k. While lowest BIC values occurred at k = 19, the 'elbow' pattern started to occur at k=8-10, with the last increase in k to reduce BIC by > 2 occurring most frequently at k=8, which we chose for further analysis. Then, 1 000 iterations of procedures described at Phillips et al (2018) were conducted, i.e., *k*-means clustering with *k* = 8 followed by DAPC (using *dapc()* function in *adegenet*) with 30 PCs (95.7% of variance) and four discriminant functions (99% of variance), the *optim.a.score()* procedure of *adegenet*, which aims to minimise overfitting and a repeat of DAPC with the number of PCs returned by *optim.a.score()*. Names of clusters assigned by the second DAPC were then standardized between runs and assigned each sequence to its model supertype.

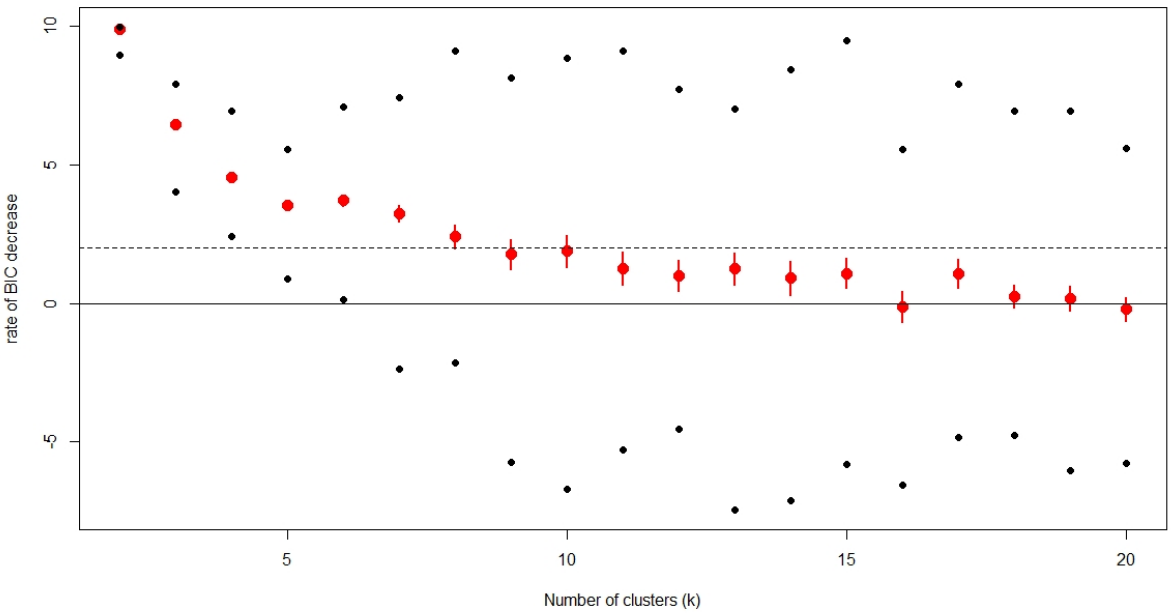

Figure. Change in BIC ($\Delta BIC = BIC_k - BIC_{k+1}$) with additional clusters. Mean values (large red points), mean±CI (vertical red lines) and range (small black points) calculated from 100 iterations are shown. Solid line at $\Delta BIC = 0$, dashed line at $\Delta BIC = 2$.

References

Jombart, T. adegenet: a R package for the multivariate analysis of genetic markers. *Bioinformatics* **24**, 1403-1405 (2008).

Osorio, D., Rondon-Villarreal, P. & Torres, R. Peptides: A package for data mining of antimicrobial peptides. *The R Journal* **7**, 4-14 (2015).

Sandberg, M., Eriksson, L., Jonsson, J., Sjöström, M. & Wold, S. New chemical descriptors relevant 47 for the design of biologically active peptides. A multivariate characterization of 87 amino acids. *J Med Chem* **41**, 2481-2491 (1998).
